# Consistent downregulation of the cleft lip/palate-associated genes *IRF6* and *GRHL3* in carcinomas

**DOI:** 10.3389/fonc.2022.1023072

**Published:** 2022-11-15

**Authors:** Ludovica Parisi, Carolin Mockenhaupt, Silvia Rihs, Farah Mansour, Christos Katsaros, Martin Degen

**Affiliations:** Laboratory for Oral Molecular Biology, Department of Orthodontics and Dentofacial Orthopedics, University of Bern, Bern, Switzerland

**Keywords:** IRF6, GRHL3, cancer, differentiation, tumor suppressor, orofacial cleft

## Abstract

Interferon Regulatory Factor 6 (IRF6) and Grainyhead Like Transcription Factor 3 (GRHL3) are transcription factors that orchestrate gene regulatory networks required for the balance between keratinocyte differentiation and proliferation. Absence of either protein results in the lack of a normal stratified epidermis with keratinocytes failing to stop proliferating and to terminally differentiate. Numerous pathological variants within *IRF6* and *GRHL3* have been identified in orofacial cleft-affected individuals and expression of the two transcription factors has been found to be often dysregulated in cancers. However, whether orofacial cleft-associated *IRF6* and *GRHL3* variants in patients might also affect their cancer risk later in life, is not clear yet. The fact that the role of IRF6 and GRHL3 in cancer remains controversial makes this question even more challenging. Some studies identified IRF6 and GRHL3 as oncogenes, while others could attribute tumor suppressive functions to them. Trying to solve this apparent conundrum, we herein aimed to characterize IRF6 and GRHL3 function in various types of carcinomas. We screened multiple cancer and normal cell lines for their expression, and subsequently proceeded with functional assays in cancer cell lines. Our data uncovered consistent downregulation of IRF6 and *GRHL3* in all types of carcinomas analyzed. Reduced levels of IRF6 and *GRHL3* were found to be associated with several tumorigenic properties, such as enhanced cell proliferation, epithelial mesenchymal transition, migration and reduced differentiation capacity. Based on our findings, *IRF6* and *GRHL3* can be considered as tumor suppressor genes in various carcinomas, which makes them potential common etiological factors for cancer and CLP in a fraction of CLP-affected patients.

## Introduction

When epithelial cells accumulate somatic mutations within oncogenic “driver genes” and other genetic alterations, they can give rise to carcinomas. Carcinomas can be divided into adenocarcinomas, squamous cell carcinomas (SCC), transitional carcinomas and basal cell carcinomas, which originate in specialized, glandular epithelial cells (e.g., breast, colon, lung), in the squamous epithelia (e.g., skin, mucosa), in the epithelia of the urinary system (e.g., bladder, kidney), and basal cells present in the deepest layer of the epidermis (e.g. skin), respectively. A common hallmark of aggressive carcinomas is that they gain mesenchymal properties while losing epithelial characteristics through a process called epithelial-mesenchymal transition (EMT), which is associated with a more invasive phenotype (1). Consequently, carcinomas often contain immature and poorly differentiated epithelial cells, and tend to have a worse clinical outcome and prognosis than well-differentiated cancers (2–4). Accordingly, the histological assessment of the differentiation status of carcinomas is important as it can provide crucial insights into the tumor etiology and its aggressiveness, prognosis, and response to treatment (5, 6).

Interferon Regulatory Factor 6 (IRF6) and Grainyhead Like Transcription Factor 3 (GRHL3) are two transcription factors, which regulate the balance between keratinocyte proliferation and differentiation. Consistently, the phenotypes of *Irf6*- and *Grhl3*-deficient mice are highly similar to each other both being lethal, displaying a thick hyperproliferative epidermis and terminal differentiation defects leading to lack of a proper skin barrier (7–9). In addition, IRF6 and GRHL3 are known to be crucial regulators of craniofacial development as both are required for the formation and maintenance of the periderm. The periderm is a transient layer of stratified primitive ectoderm-derived cells that covers the developing epithelia, acts as a barrier, and prevents orofacial cleft-causing epithelial fusions between the palate and the mandible and/or the tongue (10, 11). Consequently, many *IRF6* and *GRHL3* variants, leading to haploinsufficiency (12) and dominant-negative effects (13), respectively, have been identified in individuals affected by either non-syndromic cleft lip/palate (CLP) or Van der Woude Syndrome (VWS) and Popliteal Pterygium Syndrome, two autosomal dominant conditions characterized by CLP (13–16).

Genes that are indispensable for embryogenesis may play essential roles for cancer growth and survival later in adults (17). Fittingly, *IRF6* and *GRHL3* are frequently found dysregulated in carcinomas (18, 19). Although the role of IRF6 and GRHL3 in carcinomas is not fully elucidated yet, it is plausible to link their expression to the differentiation status of carcinomas. However, numerous conflicting and controversial data exist on their tumor-specific expression and function, even in studies analyzing the same cancer tissues. While some reports point towards oncogenic functions for IRF6 and/or GRHL3, others describe them as tumor suppressors. Notably, the latter function would fit to their physiological role during development as guardians of epithelial homeostasis (19, 20). Furthermore, reduced IRF6 levels in tumors reflect the situation in those VWS patients, who harbor *IRF6* variants leading to haploinsufficiency of IRF6 and to terminal differentiation defects in keratinocytes (12, 21, 22). This observation is intriguing as some population-based studies have indicated an increased cancer risk in CLP-affected individuals and unaffected first-degree relatives (23). However, this association, which might suggest a shared genetic etiology between CLP and cancer, remains controversial and inconsistent (24–27).

In the current study, we aimed to clarify the role of IRF6 and GRHL3 in various human carcinomas. Our results show that their expression is highly epithelial cell-specific, but that their levels in carcinomas are significantly decreased in all the cancer types analyzed compared to control. These results in combination with functional IRF6 and GRHL3 studies in cancer cell lines allowed us to describe both transcription factors as tumor suppressors. Therefore, it can be speculated that IRF6 and GRHL3 are genetic risk factors associated with the comorbidity of cancer and CLP.

## Materials and methods

### Expression profiling using public database

The datasets for gene and protein profiling were obtained from the Gene Expression Omnibus (www.ncbi.nlm.nih.gov/geo/) and the Human Protein Atlas (HPA; www.proteinatlas.org). The HPA was also used for the single cell expression analysis.

### Quantitative real-time polymerase chain reaction

Total RNA was extracted using the innuPREP RNA Mini kit (Analytic Jena AG, Jena, Germany) and 500 ng of RNA were used for the synthesis of cDNA with Oligo(dT)_15_ primers and the M-MLV Reverse Transcriptase (both from Promega, Dübendorf, Switzerland). Gene expression was analyzed by qPCR using the GoTaq^®^ qPCR Master Mix (Promega) on a QuantStudio 3 instrument (Applied Biosystems, Thermo Fisher Scientific, Waltham, MA, USA). Normalized expression was calculated applying the dC*_T_* method for absolute mRNA levels, or the ddC*_T_* method for relative mRNA levels. Sequences of the qPCR primers are reported in **Supplementary Table 1**.

Total healthy tissue RNA included: human lung (ATR1234152-50), cerebral cortex (A803146), mammary gland (B610021), colon (R1234090-50), transverse colon (R1234096-10-B402251, all from BioChain, amsbio, Abingdon, UK), adult brain adult (#636530), fetal brain (#636526) and cerebellum (#636535, Clontech, Takara Bio Inc, Kusatsu, Japan).

### Immunohistochemistry

Tissue microarrays (#MC245c and MC246b, amsbio) were baked for 1 h at 60°C before deparaffinization and rehydration through xylene, ethanol, and deionized (dd) H_2_O. Tris/EDTA buffer, pH 9.0 at 95°C for 30 min was used for antigen retrieval. Specific binding of primary rabbit polyclonal anti-IRF6 antibody (NBP2-49383, Novus Biologicals, Centennial, CO, USA) was visualized with the ImmPACT DAB Peroxidase Substrate (Vector Laboratories, Newark, CA, USA). Nuclei were counterstained with hematoxylin and slides mounted with Aquatex (Sigma-Aldrich, St. Louis, MO, USA).

### Cell culture

Patient-derived epithelial and mesenchymal cells were isolated from biopsies using the explant culture technique (21, 28) and cultured in Keratinocyte Serum-Free Medium (KSFM) containing 25 µg/ml bovine pituitary extract, 0.2 ng/ml epidermal growth factor, 0.4 mM CaCl_2_ and 1X PenStrep as described previously (29) and in Dulbecco’s Modified Eagle’s Medium (DMEM) supplemented with 10% FCS and 1X Pen/Strep, respectively. To maintain healthy high-density keratinocyte cultures, they were re-fed daily with a 1:1 medium (1:1 vol/vol Ca^2+^-free DMEM with completely supplemented KSFM) as reported elsewhere (30). All the cells and their media and supplements (Thermo Fisher Scientific) used in this study are listed in **Supplementary Table 2**. Live images of the cells were taken with a Leica DMIL LED inverted microscope (Leica Biosystems, Nussloch, Germany).

### Immunoblotting

Cell extracts were prepared in 1X RIPA buffer (10 mM Tris-HCl pH 8.0, 1 mM EDTA, 0.1% Na deoxycholate, 0.1% SDS, 1% NP40, 140 mM NaCl) supplemented with cOmplete Mini™ Protease Inhibitor cocktail and PhoSTOP EASYpack (both from Sigma-Aldrich). Protein concentrations were measured using a Bicinchoninic acid assay (Pierce, Thermo Fisher Scientific) following the instructions. Ten µg of total protein in sample loading buffer (62.6 mM Tris-HCl pH 6.8, 2% SDS, 10% glycerol, 0.01% bromophenol blue) containing 100 mM dithiothreitol (Sigma Aldrich) were boiled for 5 min at 95°C and separated under reducing conditions by SDS-PAGE. Thereafter, proteins were blotted onto nitrocellulose membranes (Sigma-Aldrich), which were subsequently stained with 0.1% amido black solution (Merck, Burlington, MA, USA) to control for equal protein loading and blotting efficiency. Membranes were washed in Tris-Buffered Saline (TBS) pH 7.4, containing 0.05% Tween-20 (TBS-T), blocked in 5% skim milk in TBS-T, incubated with primary antibody overnight at 4°C, washed in TBS-T and incubated with horseradish peroxidase-conjugated anti-mouse/rabbit IgGs. Blots were developed using SuperSignal West Pico or Dura solutions (Pierce, Thermo Fisher Scientific) and scanned with an Imager Chemi Premium Instrument (VWR, Darmstadt, Germany). Some immunoblots were densitometrically quantified using the free software ImageJ available at https://imagej.nih.gov/ij/download.html.

Primary antibodies used: rabbit polyclonal anti-E-Cadherin (20874-1-AP), anti-N-Cadherin (22018-1-AP), and anti-ß-actin (20536-1-AP, all from Proteintech, Manchester, UK), and anti-GRHL3 (ARP33196_P050, Aviva Systems Biology Corporation, San Diego, CA, USA) as well as mouse monoclonal anti-IRF6 (14B2C16, BioLegend, San Diego, CA, USA), anti-Vimentin (clone VI-10, A86652, Antibodies.com, Cambridge, UK), and anti-Vinculin (clone hVIN-1, V9131, Sigma-Aldrich).

### Immunofluorescence

Cells were fixed in 4% paraformaldehyde (Grogg Chemie, Stettlen, Switzerland), rinsed in Phosphate-Buffered Saline (PBS), permeabilized with 0.1% Triton-X-100 (Sigma-Aldrich) for 5 min, blocked in 3% Bovine Serum Albumin (Sigma-Aldrich) for 30 min and incubated with primary antibodies for 2 h at room-temperature (RT). Afterwards, cultures were washed in PBS, incubated with fluorescently labeled secondary antibodies (Molecular Probes, Thermo Fisher Scientific) or with phalloidin (Sigma-Aldrich) for 1 h at RT in the dark. Incubation was followed by washes in PBS and one final rinse in ddH_2_O before being coverslip-mounted using the Vectashield Mounting Medium containing DAPI (Vector Laboratories). Samples were analyzed under an Olympus BX-51 phase microscope equipped with fluorescence filters U-MWIBA3 for Alexa Fluor 488, U-MWIGA3 for Alexa Fluor 568, and U-MNUA2 for DAPI (Olympus Life Science Solution, Tokyo, Japan).

For the quantification of Keratin10-positive cells, 10 random microscopic fields were chosen with a total number of 450 cells per field. Keratin10 quantification was performed with ImageJ.

Primary antibodies used: rabbit polyclonal, anti-IRF6 (NBP2-49383), anti-TGM1 (NBP2-34062, both from Novus Biologicals), anti-Loricrin (PA5-30583, Thermo Fisher Scientific), and anti-Filaggrin (orb10662, biorbyt, Cambridge, UK) as well as mouse monoclonal anti-Involucrin (clone SY5, BioRad, Hercules, Ca, USA) and anti-Keratin10 (DE-K10, Thermo Fisher Scientific).

### Generation of IRF6/GRHL3 overexpressing cells

*IRF6* and *GRHL3* cDNAs in the lentiviral expression vector CAD-IRES-GFP were purchased from Genescript (Leiden, Netherlands). Lentiviral particles were produced in HEK293T cells with the packaging vectors psPAX2 and pMD2.G (both gifts from Didier Trono (Addgene plasmids #12260 and 12259)). Twenty-four h after cell transfection using ViaFect (Promega) in OptiMEM (Thermo Fisher Scientific), transfection medium was replaced with DMEM containing 10 mM HEPES, pH 7.4. Viral particles were collected 48 and 72 h after transfection, pooled, sterile-filtered and used for transducing SCC-68 and T47D cell lines in the presence of 5 μg/ml polybrene (Sigma-Aldrich). Transduced cells were sorted for GFP using the MoFlo Astrios™ EQ cell sorter (Beckman Coulter Life Sciences, Krefeld, Germany) and the GFP^+^ population was used for rescue experiments.

### Cell growth

Proliferation was determined by cell counting using standard techniques. Briefly, 10^5^ cells were seeded into 10 cm culture dishes. One dish was counted after initial attachment (day 0) for normalization, and parallel cultures on day 3 and 5 using a Neubauer Counting chamber.

### Migration analysis

Monolayers were scratched in 96-well plates using sterile 20 μl pipette tips. Afterwards, cells were washed with PBS and replenished with fresh medium. Images were acquired with the IncuCyte S3 live imaging device (Sartorius, Göttingen, Germany). Scratch assays were quantified using ImageJ. Videos of cell migration were created with pictures of cells acquired at 0, 2, 4, 6, 8 and 10 h at a frame rate of 2 frames per second (fps) using ImageJ.

### Literature search

A literature search was performed on the knowledge of IRF6 and GRHL3 in cancer using the databases Medline (PubMed (https://pubmed.ncbi.nlm.nih.gov) and Embase (www.embase.com). Central for the search was the following focus question: What is the role and function of the transcription factors IRF6 and GRHL3 in cancer? The search strategy is shown in **Supplementary Table 3**. The literature search was conducted on March 29, 2022.

### Statistics

Experiments were performed at least three times in multiple replicates. Data were analyzed using Prism 7 (GraphPad, La Jolla, CA, USA). Data are reported as means ± standard deviation (SD). Multiple comparisons were performed using one- or two-way analysis of variance (ANOVA) with Tukey’s *post hoc* test. Data were considered significant when p<0.05.

## Results

### IRF6 and *GRHL3* are specifically expressed in epithelial cells

**Figure 1:**
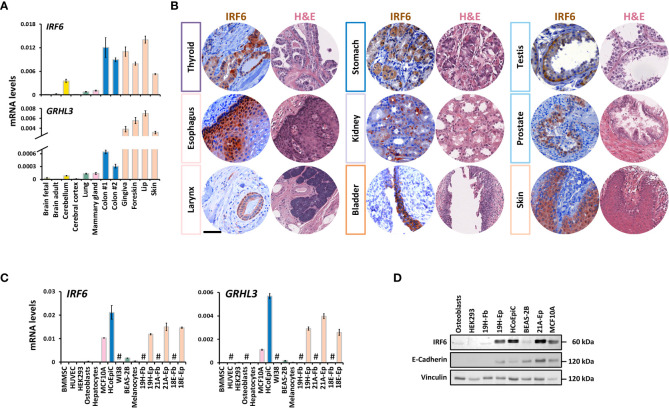
IRF6 and *GRHL3* expression in healthy human adult tissues and cells **(A)** QPCR analysis of *IRF6* and *GRHL3* in a panel of healthy human adult tissues. Color code refers to tissue groups introduced in **Supplementary Figure 1A**. **(B)** IHC for IRF6 as well as a representative H&E staining of a set of normal human tissues. Note that IRF6 exhibits nuclear as well as cytoplasmic localization. Scale Bar: 50 µm. **(C)** QPCR analysis of *IRF6* and *GRHL3* in a panel of healthy human cell strains/lines. not detectable (cT > 32) **(D)** Immunoblot for the proteins IRF6 and E-Cadherin in a set of human cell strains/lines. Note the typical IRF6 double-band indicative of its phosphorylated and non-phosphorylated form, respectively. Full-length blots are shown in **Supplementary Figure 6** kDa, kilo Daltons.

IRF6 and GRHL3 are believed to be epithelial-specific transcription factors. However, their expression in non-epithelial cells has also been described. IRF6 is expressed in certain immune cells, neurons, pre-and mature adipocytes, and osteocytes (31–34), while GRHL3 can be detected in brain cells during development, endothelial cells, and in limb bud progenitor cells that contribute to bone development and bone repair (35–37). To shed more light on the expression of *IRF6* and *GRHL*3 in healthy human adult tissues, we consulted the Human Protein Atlas (HPA) and the GEO database. Data from the HPA revealed a broad *IRF6* expression among various tissues, with skin and esophagus being the most prominent sources of *IRF6* (**Supplementary Figure 1A**). In contrast, *GRHL3* expression was more restricted with only a handful of tissues being robustly positive. Identical to *IRF6*, highest *GRHL3* levels were found in skin and esophagus (**Supplementary Figure 1A**). Analysis of the GSE14938 dataset available on the GEO database identified the skin, mammary gland, and trachea as tissues with the highest RNA levels of *IRF6* and *GRHL3* (**Supplementary Figure 1B**). Next to gene datasets, we also screened a panel of healthy human tissues for *IRF6* and *GRHL3* transcripts by qPCR. High levels of *IRF6* and *GRHL3* were detected in skin-, and oral-derived epithelial tissues as well as in colon, while moderate to low levels were found in mammary gland, lung, and cerebellum (**Figure 1A**). These results were in good agreement with the HPA and GEO datasets. As both transcription factors seem to have their major function in epithelial cells during development and as *GRHL3* is a direct IRF6 target gene (38), we wondered whether *IRF6*, *GRHL3*, and the epithelial marker E-Cadherin (*CDH1*) correlated in the investigated cohorts. Indeed, there was a positive correlation between *IRF6* and *GRHL3*, which both matched up with *CDH1*, but not with the mesenchymal marker Vimentin (*VIM*) (**Supplementary Figure 1C**).

Analysis of human tissue microarrays allowed us to verify IRF6 expression on protein level by IHC in various healthy tissues. IRF6 was robustly expressed in the thyroid gland, esophagus, larynx, stomach, kidney, bladder, testis, prostate, and skin (**Figure 1B**), while it was only weakly detectable or even absent in pancreas, lymph node, and skeletal muscle (**Supplementary Figure 1D**). These results were in agreement with the *IRF6* mRNA data. In all positive tissues, IRF6 was mostly localized in the cytoplasm, but could also be detected in the nucleus.

Next, we wanted to elucidate the specific cell types responsible for *IRF6* and *GRHL3* expression in various tissues. Therefore, we explored the “Single Cell information” based on single cell RNA sequencing data from breast, lung, colon, and skin tissues available at the HPA. We focused on these four tissues since they all contained detectable amounts of *IRF6* and *GRHL3* (**Figure 1A**). All four tissues showed highest expression of *IRF6* and *GRHL3* in epithelial cells: Glandular epithelial cells in the breast, glandular and specialized epithelial cells in the lung, intestinal goblet cells and enterocytes in the colon as well as basal and suprabasal keratinocytes in the skin (**Supplementary Figure 2A**). We further assessed these findings in a set of normal human cells by qPCR (**Figure 1C**) and immunoblotting (**Figure 1D**). In accordance with the single cell data, IRF6 and *GRHL3* were mostly detectable in E-Cadherin (*CDH1*)-positive epithelial cells and there was a positive correlation between the three genes (**Supplementary Figure 2B**). Besides epithelial cells, *IRF6* and *GRHL3* were only detectable in melanocytes (**Figure 1C**), which also expressed relatively high *CDH1* (data not shown). Collectively, we report that IRF6 and *GRHL3* are highly epithelial cell-specific.

### IRF6 and *GRHL3* are downregulated in cancer cell lines and tissues

**Figure 2:**
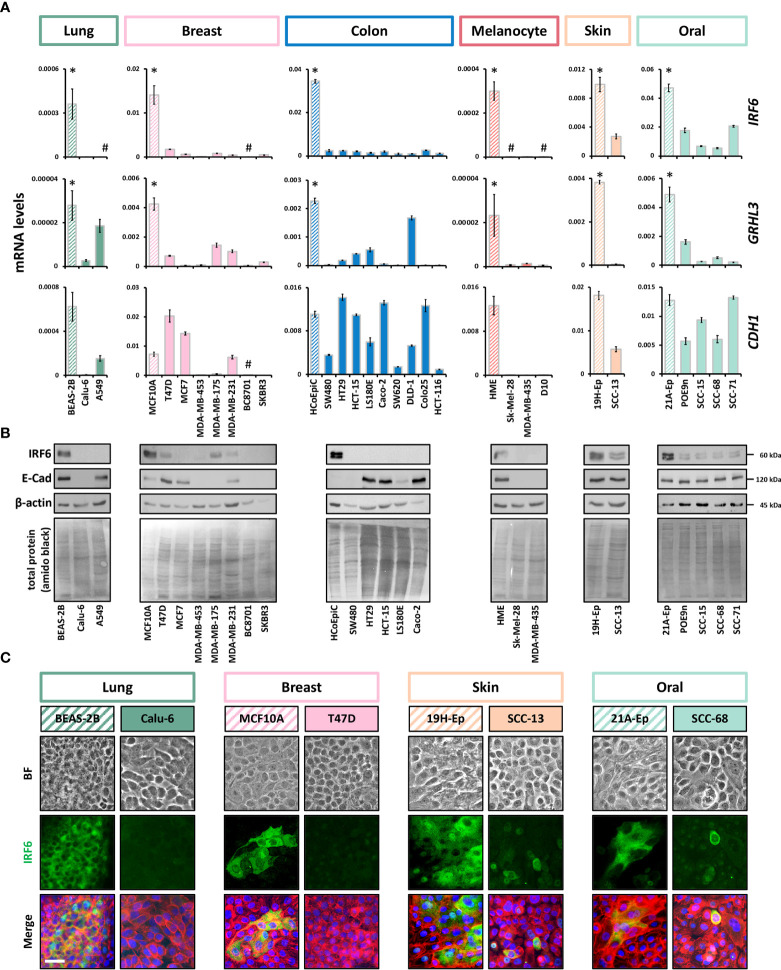
IRF6 and *GRHL3* downregulation in cancer cell lines **(A)** Panels of cancer cell lines and corresponding normal cells (striped boxes) were screened for *IRF6*, *GRHL3*, and *CDH1*. Note that RNA was extracted from all cell cultures at a cell density of approximately 70% confluency kDa, kilo Daltons. * p < 0.05 control vs. cancer cell lines; # not detectable (c*_T_* > 32). HME: human melanocytes. **(B)** Immunoblots for IRF6 and E-Cadherin in a subset of the analyzed cancer cell lines and controls. Full-length blots are shown in **Supplementary Figure 6**. HME: human melanocytes. **(C)** Brightfield (BF) pictures, IRF6 staining, as well as the merge of IRF6 (green) with actin (red) and cell nuclei (blue) is shown for breast and lung cancer, skin and oral SCC samples and their corresponding normal controls. Note that IRF6 is predominantly expressed in the cytoplasm of the various cells. Scale bar: 25 µm.

To gain a better overview of published results on IRF6 and GRHL3 expression and function in cancer, we performed a literature search (**Supplementary Table 4**). While for certain cancer types (e.g., skin cancer) the roles of IRF6 and GRHL3 were found to be consistent (tumor suppressors), the findings were more controversial in others (e.g., female tissues). To clarify these apparent discrepancies on the role of IRF6 and GRHL3 in cancer, we screened breast, colon, lung, and skin cancer cell lines for *IRF6*, *GRHL3*, and *CDH1* transcripts and compared their levels to them in corresponding control cell lines by qPCR. We observed a consistent and significant downregulation of both *IRF6* and *GRHL3* in all the cancer cell lines analyzed compared to controls (**Figure 2A**). However, there was no evidence of a positive correlation between *IRF6*, *GRHL3*, and *CDH1* in cancer cells, as had been previously observed in healthy tissues and cells (**Supplementary Figure 1C** and **Supplementary** **Figure 2B**). We were further able to confirm cancer cell-specific IRF6 protein downregulation by immunoblotting (**Figure 2B**) and IF staining (**Figure 2C**) in all those cancer types we had normal control cells available (i.e., lung, breast, skin, and oral cavity). A combination of data extracted from the HPA with our own IHC analysis on representative normal/cancer tissues supported our finding of reduced IRF6 levels in cancer tissues compared to controls (**Supplementary Figure 3A**). We also screened a panel of brain cancer cell lines for *IRF6* and *GRHL3* (**Supplementary Figure 3B**). *IRF6* was not detectable in 4/5 brain cancer cell lines tested, while *GRHL3* levels were variable, ranging from not detectable (e.g., Hs683, T98G) to high expression in LN319. Since a normal human brain cell line was not available, we used normal brain tissue (see **Figure 1A**) as a control, which made a comparative analysis not feasible. However, we obtained further evidence of reduced IRF6 levels in brain cancer compared to control by the HPA database and our own IHC analysis (**Supplementary Figure 3C**).

### Re-expression of IRF6 and GRHL3 in cancer cell lines suppresses certain tumor traits

**Figure 3:**
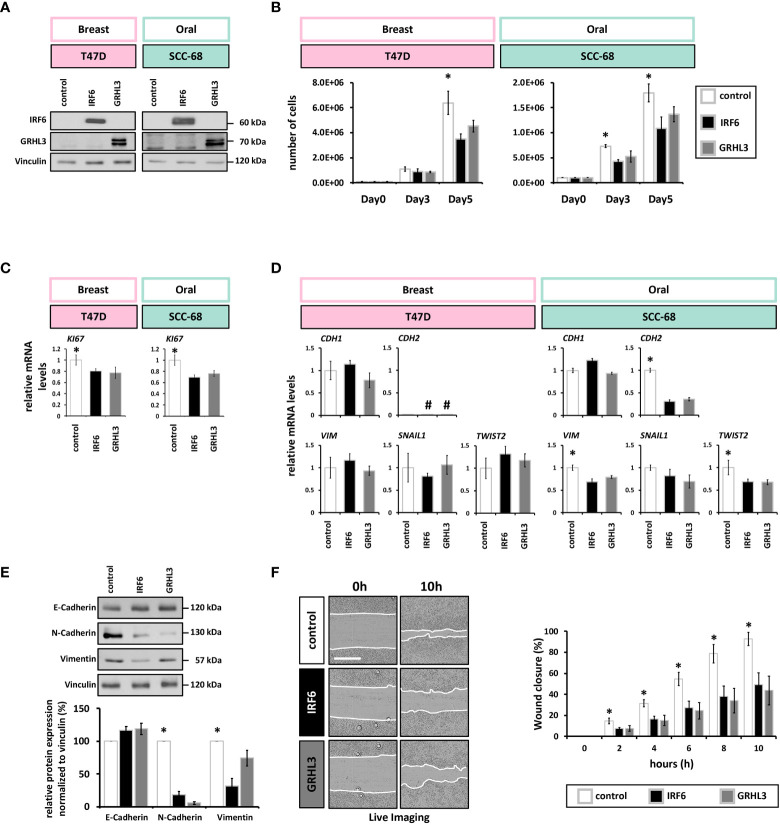
IRF6 and GRHL3 overexpression impairs cancer cell proliferation and migration **(A)** Immunoblots for IRF6 and GRHL3 confirming their overexpression. Full-length blots are shown in **Supplementary Figure 6**. kDa, kilo Daltons. **(B)** Cell growth of empty vector (white)-, IRF6 (black)-, and GRHL3 (gray)- transduced T47D (left) and SCC-68 cell lines (right) shows reduced number of cells by the enhanced presence of IRF6 and GRHL3. * p < 0.05 control vs. IRF6 and GRHL3 **(C)** qPCR analysis of the proliferation marker *KI67* in transduced T47D and SCC-68 cell lines. * p < 0.05 control vs. IRF6 and GRHL3. **(D)** Transduced cells were analyzed for the expression of EMT markers *CDH1*, *CDH2*, *VIM*, *SNAIL1*, *TWIST2*. not detectable (cT > 32). **(E)** E-Cadherin, N-Cadherin, and Vimentin were analyzed by immunoblots in control and IRF6 and GRHL3 transduced SCC-68 cells. Quantification of the blots is shown below the immunoblots. * p < 0.05 control vs. IRF6 and GRHL3 for N-Cadherin and Vimentin. Full-length blots are shown in **Supplementary Figure 6**. kDa, kilo Daltons. **(F)** Representative live imaging pictures at t=0 h (left) and t=10 h (right) after scratching SCC68 control, SCC68/IRF6, and SCC68/GRHL3. White lanes indicate the migrating fronts on each side of the cell-free gap (left). Quantification of the closure of the cell-free gap is shown on the right. Note a significantly reduced migratory behavior of SCC-68/IRF6 and SCC-68/GRHL3 compared to control starting from t=2 h * p < 0.05 control vs. IRF6 and GRHL3. Scale bar: 500 µm. Movies of the various cell lines are shown in **Supplementary Movies 1–3**.

To address the consequences of low IRF6 and GRHL3 levels in cancer cell lines, we rescued their expression in the breast cancer cell line T47D and in the oral squamous carcinoma cell line SCC-68 (**Figure 3A** and **Supplementary Figure 4**). Overexpression of both IRF6 and GRHL3 significantly slowed-down the proliferation rate of T47D and SCC-68 cells as assessed by cell counting (**Figure 3B**) and determining *KI67* transcript levels (**Figure 3C**).

Both IRF6 and GRHL3 have been found to be implicated in regulating EMT during development as well as in cancer progression (39–41). Therefore, we wished to know whether elevated IRF6 and GRHL3 levels in T47D and SCC-68 were able to modulate the expression levels of typical EMT-related markers. In T47D, overexpression of neither IRF6 nor GRHL3 influenced the transcript levels of *CDH1*, N-Cadherin (*CDH2)*, *VIM*, *SNAIL1* or *TWIST2* (**Figure 3D**). In contrast, IRF6 and GRHL3 overexpression in SCC-68 significantly reduced levels of the mesenchymal markers N-Cadherin, VIM, and *TWIST2* while none of the transcription factors was able to significantly induce E-Cadherin (**Figure 3D, E** **)**.

The fact that EMT is associated with an increased migration prompted us to analyze the migratory behavior of SCC-68 transduced with either IRF6 or GRHL3. Therefore, we employed scratch assays and monitored the closure of the cell-free gap. When compared to control, the SCC-68 cell lines overexpressing IRF6 or GRHL3 significantly delayed the closing of the scratch (**Figure 3F**), which fits to the partially reversed EMT phenotype in these cell lines (SCC-68/IRF6 and SCC-68/GRHL3). However, we did not observe differences in the migration pattern of the cells moving into the open space among the different cell lines (**Supplementary Movies 1–3**).

**Figure 4:**
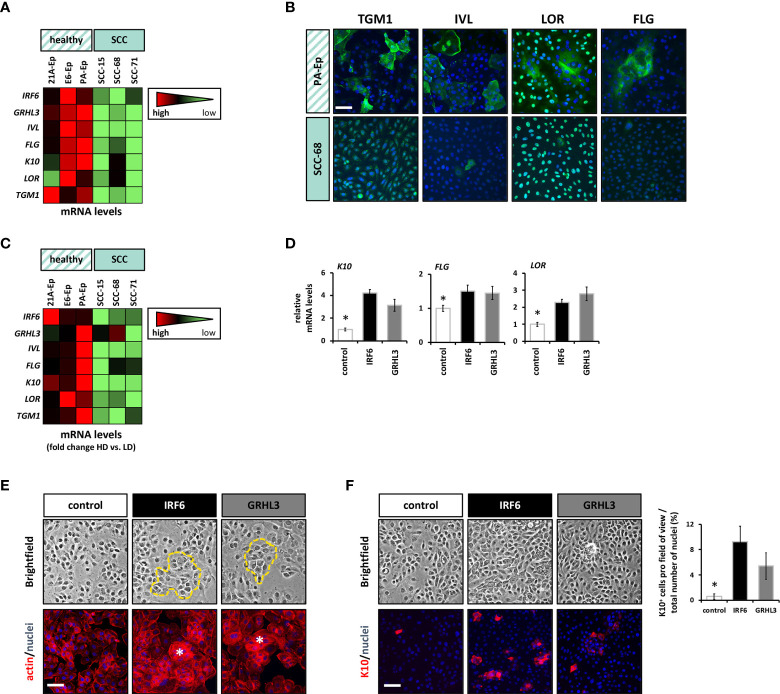
Overexpression of IRF6 and GRHL3 induces differentiation. **(A)** The differentiation status of three normal cell strains compared to three SCC lines was assessed by qPCR for a panel of differentiation markers (*IRF6*, *GRHL3*, *IVL*, *FLG*, *K10*, *LOR*, and *TGM1*). The data are shown as a heatmap. Scale: light green – low gene expression; red – high gene expression. **(B)** IF staining for TGM1, IVL, LOR, and FLG in PA-Ep (non-cancerous) vs. SCC-68 indicates less differentiated cells in the SCC-68 cultures. Scale bar: 50 µm. Note that staining for LOR and TGM1 resulted in a nuclear background staining (**Supplementary Figure 5**). **(C)** The same healthy cell strains and SCC cell lines were analyzed in low density (LD) and their corresponding high density (HD) cultures, and the same set of differentiation genes was analyzed by qPCR. The results are shown as heatmap of the fold inductions (HD vs. LD). Scale: light green – low gene induction; red – high gene induction. **(D)** The effect of forced expression of IRF6 (black) and GRHL3 (gray) compared to control SCC-68 (white) on differentiation markers (*K10*, *FLG*, and *LOR*) was determined by qPCR. * p < 0.05 control vs. IRF6 and GRHL3. **(E)** Brightfield pictures and corresponding pictures with actin staining indicate the presence of differentiating cell groups (dashed yellow line in the brightfield images and asterisks in the actin staining) in SCC68 cells transduced with IRF6 or GRHL3. Scale bar: 50 µm. **(F)** K10 staining in dense cultures of transduced SCC-68 cells (left) and its quantification (right). Note the presence of significantly more K10-positive cells in SCC-68/IRF6 and SCC-68/GRHL3 compared to SCC-68 control. Scale bar: 50 µm. * p < 0.05 control vs. IRF6 and GRHL3.

As both IRF6 and GRHL3 are key regulators of the proliferation-differentiation balance of keratinocytes (7, 8), we wanted to explore the possibility that reduced IRF6 and GRHL3 levels in SCC cell lines correlate with a defective differentiation potential of SCC cells. Initially, we compared the levels of typical differentiation markers at high cell density (HD) in three non-cancerous epithelial cell strains and three SCC cell lines. All SCC cell lines displayed reduced *IRF6* and *GRHL3* levels, which were associated with lower levels of Involucrin (*IVL*), Filaggrin (*FLG*), Keratin10 (*K10*), Loricrin (*LOR*), and Transglutaminase1 (*TGM1*) compared to controls (**Figure 4A**). IF staining for the differentiation markers TGM1, IVL, LOR, and FLG confirmed the finding of differentiation defects in SCC cells as all proteins showed significant lower expression in SCC-68 when compared to PA-Ep (**Figure 4B**). Being aware that different tissue donors might account for the observed variations in the differentiation markers among our cells, we further assessed each individual’s differentiation potential by determining the induction of the markers at HD compared to low cell density (LD) (**Figure 4C**). While the normal oral keratinocytes strongly induced all the tested markers upon reaching HD, the oral SCC cell lines failed to do so, which confirmed and established impaired differentiation within the SCC cell lines. Next, we tested whether overexpression of IRF6 or GRHL3 would be enough to rescue this differentiation phenotype. Overexpression of both transcription factors in SCC-68 resulted in increased levels of several differentiation markers, including *K10*, *FLG*, and *LOR* (**Figure 4D**). This observation was confirmed by brightfield imaging and IF staining for actin, which showed the presence of differentiating cell groups with signs of stratification (**Figure 4E**, dotted line (top) and asterisk (bottom)) upon forced IRF6 and GRHL3 expression. Such cells were not apparent in the control cells. Finally, we stained SCC-68 HD cultures for K10 and identified a significant increase of K10-positive SCC-68 cells upon transduction of IRF6 (9.3% K10^+^ cells) or GRHL3 (5.4% K10^+^ cells) compared to control (0.6% K10^+^ cells) (**Figure 4F**). Collectively, our functional assays indicate that rescue of IRF6 and GRHL3 in cancer cell lines has the potential to normalize parts of their tumorigenic phenotype by affecting proliferation, EMT, migration, and differentiation in a tissue-specific manner.

## Discussion

A fraction of non-syndromic and syndromic CLP patients harbors pathological *IRF6* or *GRHL3* variants that result in loss of their regular activities as transcription factors. As such, it is notable that in case of *IRF6*, identical variants have also been identified in head and neck SCCs (42, 43). Despite this discovery, the roles of IRF6 and GRHL3 in cancers remain controversial as both factors have been described as oncogenes and tumor suppressors (**Supplementary Table 4**). However, the discrepancy concerning IRF6 and GRHL3 expression and function in carcinomas is mainly derived from studies analyzing their roles in adenocarcinomas. In skin and oral cancers, IRF6 and GRHL3 have been consistently found to act as tumor suppressors. Why the expression and function of IRF6 and GRHL3 are ambiguous in adenocarcinomas and not in skin and oral cancers is not known yet. The apparent controversy on their expression and role in tumors might reflect tissue-specific activities, which warrants further studies aiming to unravel the physiological function of IRF6 and GRHL3 in various tissues in more detail, or stem from varying experimental study conditions applied (e.g., comparing cell lines grown to different cell densities).

To the best of our knowledge, this is the first report analyzing in parallel both IRF6 and *GRHL3* in several different carcinomas. Our data report a consistent downregulation of both IRF6 and *GRHL3* (**Figure 2**). The availability of POE9n, a non-tumorigenic cell line cultured from an oral dysplasia (44), allowed us to further speculate that loss of IRF6 and *GRHL3* are rather early events during oral SCC progression as POE9n expressed significantly lower amounts of IRF6 and *GRHL3* than healthy control (**Figure 2**). Although we also aimed to stain for GRHL3 by IHC, and to detect the endogenous GRHL3 protein by immunoblotting and IF staining, we were not able to do so. None of our three tested anti-GRHL3 antibodies recognized the endogenous protein and only could detect forced GRHL3 overexpression in cells (see **Figure 3A**). All our results strongly suggest that IRF6 and GRHL3 act as tumor suppressors in at least the cancer tissues we analyzed.

Next to the expression profiling, we also provide functional evidence that both *IRF6* and *GRHL3* are tumor suppressor genes: overexpression of IRF6 and GRHL3 in cancer cell lines impaired their proliferation, EMT, migration and induced their differentiation potential. Our results on reduced proliferation rate in the presence of increased levels of IRF6 and GRHL3 have been described before in cancer cell lines (20, 45). We further show that in the SCC-68 cell line EMT is dependent on the loss of IRF6 and GRHL3, which has been reported in for IRF6 nasopharyngeal SCCs as well (46). Consequently, elevated IRF6 and GRHL3 levels were able to significantly reduce the expression of the mesenchymal markers N-Cadherin, VIM, and *TWIST2* (**Figures 3D, E** **)**. The role of IRF6 in impairing EMT in cancer cells contradicts what is known about its physiological function. Overexpression of IRF6 increased SNAIL2, an important mediator of EMT, during palatogenesis in mice (40) and a more recent study in human postnatal keratinocytes revealed that EMT is dependent on IRF6, but that the epithelial cell characteristics are maintained even in the absence of IRF6 (41). In contrast, GRHL3 has been reported to play an important role during MET, the reversion of the EMT process (47), which fits to our data in cancer cells. Notably, E-Cadherin levels in SCC-68 cells did not significantly change upon IRF6 or GRHL3 rescue, although there seems to be a slight tendency for increased E-Cadherin (**Figure 3E**). Forced expression of IRF6 and GRHL3 in SCC-68 was not only associated with a partial reversal of the EMT, but also with a reduced migratory capacity as evidenced by a delay in closing of a scratch *in vitro* (**Figure 3F**). While these results are in agreement with results in other cancers for IRF6 and GRHL3 (19, 46, 48), epithelial cell migration in healing wounds as well as of normal keratinocytes seem to depend on the presence of IRF6 and GRHL3 (41, 49–51). However, we did not observe any differences in the migration pattern between SCC-68 control and IRF6 and GRHL3 overexpressing cells (**Supplementary Movies 1–3**). Such differences have been reported in Irf6^-/-^ keratinocytes and in IRF6 depleted human keratinocytes that showed less directionality (49) and preferentially moved as single cells and not as continuous epithelial cell sheet (41), respectively. In the breast cancer cell line T47D, elevated IRF6 and GRHL3 did not modulate EMT marker expression (**Figure 3D**). This can be explained by the fact that T47D is a cancer cell line that maintains high levels of epithelial markers (e.g., E-Cadherin, **Figure 2**) and low levels of mesenchymal markers, and did not undergo an EMT yet (52).

It is established that the differentiation grade in SCC’s is an important (inversely proportional) indicator of tumor size, depth and aggressiveness (53). As IRF6 is regulating the proliferation-differentiation balance of epithelial cells, it is not unexpected to see an inverse correlation of IRF6 levels with the differentiation grade of cutaneous SCCs (45, 54). In our study, we were able to show that the SCC cell lines expressed reduced levels of IRF6 and *GRHL3*, and showed a reduced differentiation potential. Overexpression of IRF6 and GRHL3 in SCC-68 partially normalized this differentiation defect and K10 was re-induced in an IRF6 and/or GRHL3-dependent way (**Figure 4**).

K10, which is restricted to post-mitotic cells in the spinous layer of the skin, has been found gradually disappearing with SCC progression and strongly correlating with the differentiation status of SCCs (55–58). Lack of *K10* results in defects in skin barrier repair upon experimental barrier disruption, skin hydration, and acid sphingomyelinase activity (59). Conditional expression of K10 in the basal cell layer of the epidermis blocked cell proliferation and prevented skin cancer (60). In contrast, a more recent study described reduced tumor formation in the absence of K10 (61). Our data provide evidence that poor SCC cell differentiation might be a consequence of reduced IRF6 and GRHL3 levels. Notable, a direct role of IRF6 in regulating K10 has been recently shown in IRF6 knock-out keratinocytes by proteomics (41). Since the differentiation status often predicts patient survival in SCCs (2), IRF6 and GRHL3 levels might represent valuable prognostic and/or differentiation-defining biomarkers in SCCs.

CLP is thought to arise from gene mutations leading to defects in either cell migration, growth, differentiation, or apoptosis during development of the secondary palate (62). Alterations of the same cellular processes are also involved in cancers and often, cancer cells reactivate developmental pathways (e.g., WNT, Notch, BMP) (63). However, the relationship between CLP and cancer is subject to debate. Some studies report a co-occurrence of CLP and certain cancer types (24, 26, 27, 64–68), while others do not provide support for common genetic factors present in both conditions (69, 70). The transcription factors *IRF6* and *GRHL3* are both CLP-associated candidate genes as well as prominent tumor suppressors as described in this *in vitro* study. The mechanisms through which IRF6 and GRHL3 inhibit tumor development is not entirely elucidated yet and might be highly tissue-dependent. But they include negative regulation of the key oncogenic signaling pathway PI3K/AKT (9, 71–73) as well as the modification of cancer stem cell properties (46). It will be of considerable importance to determine whether CLP-associated *IRF6* and/or *GRHL3* variants also affect their tumor suppressive functions *in vivo*, and whether CLP/VWS patients harboring such variants might have an increased risk to develop certain types of carcinomas later in their lives. Providing such information to future CLP patients is highly relevant and might also dictate a more thorough cancer screening program. More comprehensive studies that examine gene alterations causing CLP and other craniofacial anomalies that might predispose individuals later in life to cancer, would be welcomed.

## Data availability statement

The original contributions presented in the study are included in the article/**Supplementary Material**. Further inquiries can be directed to the corresponding author.

## Ethics statement

The studies involving human participants were reviewed and approved by Ethikkommission Kanton, Bern, Switzerland (protocol # 2017-01394). Written informed consent to participate in this study was provided by the participants’ legal guardian/next of kin.

## Author contributions

LP, CM, SR, FM, and MD performed the experiments and analyzed the data. MD wrote the first draft of the manuscript, which was critically reviewed and revised by all authors. Finally, MD and CK initiated the project, planned, coordinated and designed the experiments, and provided support throughout the project. All authors contributed to the article and approved the submitted version.

## Funding

This work was supported by a Young Researcher Grant (19-155) of the Osteology Foundation, Lucerne, Switzerland (LP)

## Acknowledgments

We would like to thank Julia Feldmann for her excellent technical assistance, several colleagues for sharing cell lines with us, as well as the surgeons Dr. med. Isabelle Schnyder (Children’s Hospital, University of Bern, Switzerland), Dr. med. Giorgio C. La Scala (Division of Pediatric Surgery, Department of Pediatrics, Geneva University Hospitals, Geneva, Switzerland), Dr. Alexandra Stähli and Prof. Anton Sculean (Department of Periodontology, School of Dental Medicine, University of Bern, Bern, Switzerland) for providing biopsies used for cell isolations. The lentiviral work could be performed in the laboratory of Prof. Stephan von Gunten, Institute of Pharmacology, University of Bern. The authors would also like to express their gratitude to the FACSLab, Department for Biomedical Research, University of Bern (Stefan Müller, PhD) for FACS sorting the cells. All SCC cell lines as well as the POE9n cell line were kindly provided by Matthew R. Ramsey, PhD, Brigham and Women’s Hospital, Department of Dermatology, Boston, MA, USA) This work was supported by a Young Researcher Grant (19-155) of the Osteology Foundation, Lucerne, Switzerland (LP).

## Conflict of interest

The authors declare that the research was conducted in the absence of any commercial or financial relationships that could be construed as a potential conflict of interest.

## Publisher’s note

All claims expressed in this article are solely those of the authors and do not necessarily represent those of their affiliated organizations, or those of the publisher, the editors and the reviewers. Any product that may be evaluated in this article, or claim that may be made by its manufacturer, is not guaranteed or endorsed by the publisher.
