# Supplementary material for: Consistent downregulation of the cleft lip/palate-associated genes IRF6 and GRHL3 in carcinomas
Source: Front Oncol. 2022 Nov 15;12:1023072. doi: 10.3389/fonc.2022.1023072 (PMC9706198; doi:10.3389/fonc.2022.1023072)
Supplement: Supplementary file 1 [file DataSheet_1.pdf]

Supplementary Material

Consistent downregulation of the cleft lip/palate-associated genes *IRF6* and *GRHL3* in carcinomas

L. Parisi[1], C. Mockenhaupt[1], S. Rihs[1], F. Mansour[1], C. Katsaros[1], M. Degen[1,*]

[1] Laboratory for Oral Molecular Biology, Department of Orthodontics and Dentofacial Orthopedics, University of Bern, Bern, Switzerland

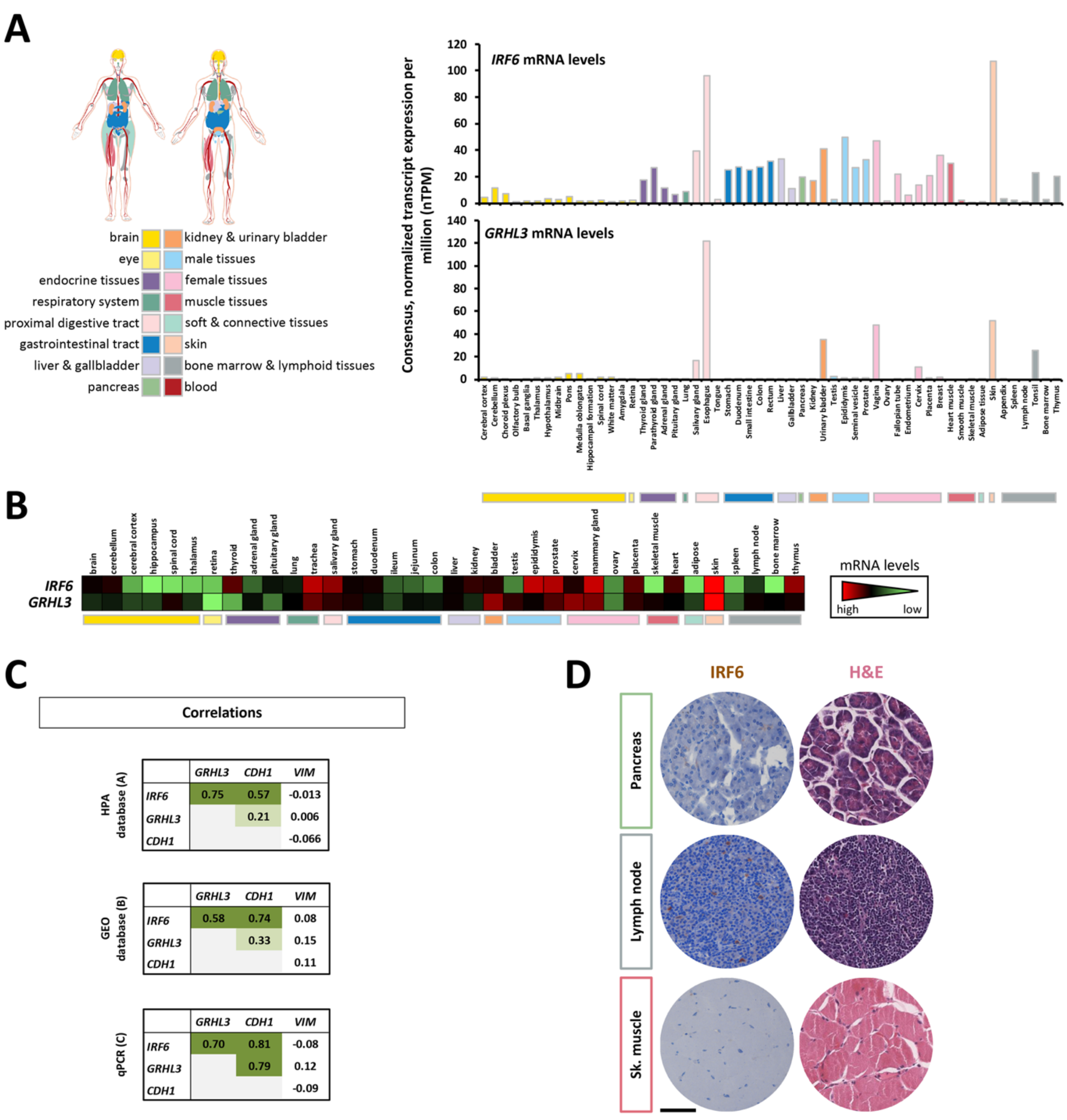

Supplementary Figure 1: IRF6 expression in normal tissues and gene correlations

(A) Consensus *IRF6* and *GRHL3* RNA expression overview from data generated from two different sources: HPA and Genotype-Tissue Expression (GTEx) RNAseq data. Color coding is based on tissue groups and is shown to the left. Data were extracted from the Human Protein Atlas (HPA). **(B)** *IRF6* and *GRHL3* expression in human adult tissues using GSE14938. Scale: light green – low gene expression; red – high gene expression. **(C)** Correlations of *IRF6*, *GRHL3*, *CDH1*, and *VIM* within the various gene expression profiling data sets analyzed in **Figure 1A and Supplementary Figure 1A, B**. Shown is the Pearson's Correlation Coefficient. Dark green: strong positive correlation; Light green: positive correlation. **(D)** Tissue of normal human pancreas, lymph node, and skeletal (sk.) muscle were stained with IRF6 (left). All three tissue do not express detectable levels of IRF6. Corresponding H&E staining of the tissues is shown on the right. Scale Bar: 50 µm.

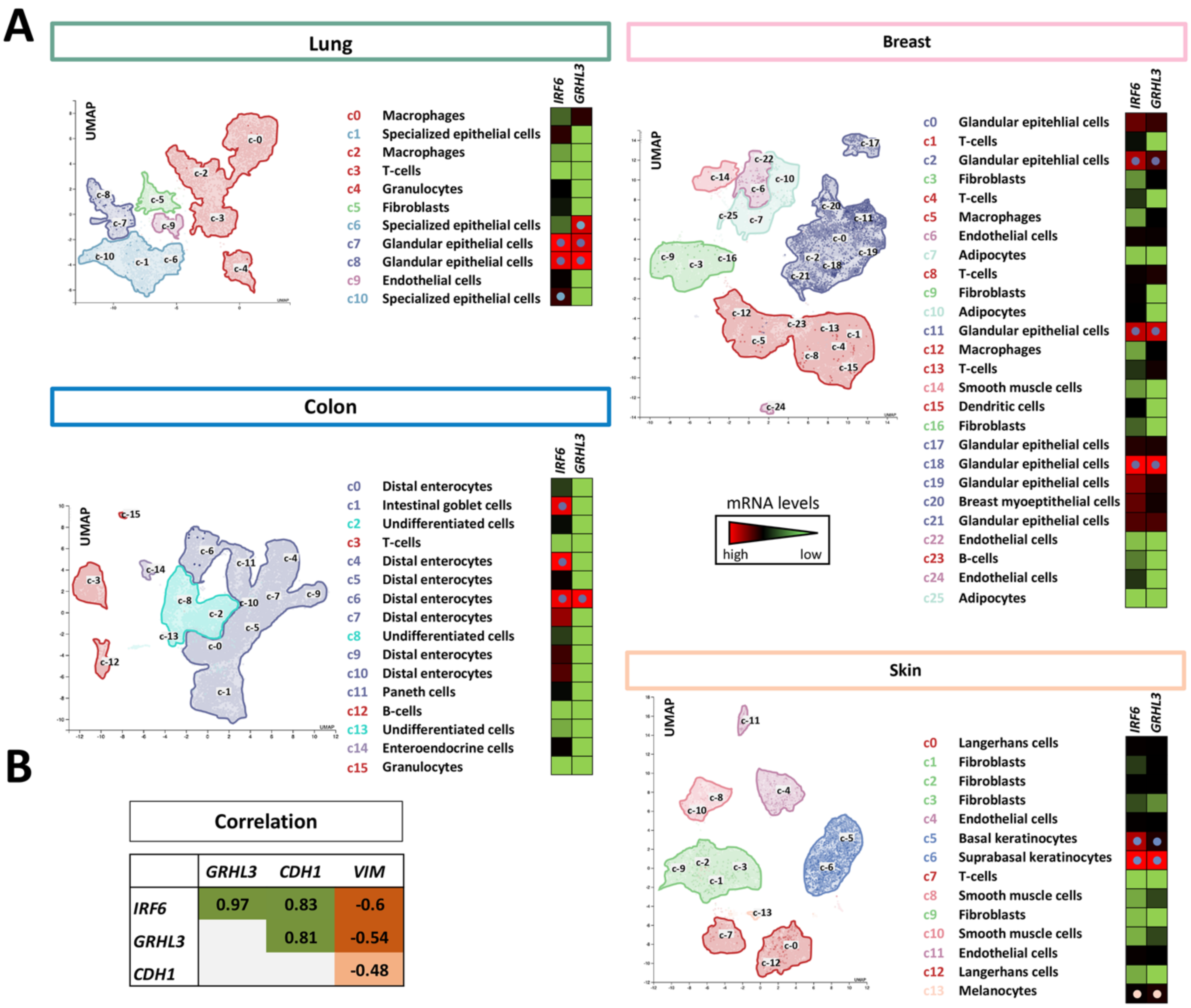

Supplementary Figure 2: Specific expression of *IRF6* and *GRHL3*, and positive correlation of *IRF6*, *GRHL3*, and *CDH1* in normal epithelial cells

(A) Single cell RNA expression levels of *IRF6* and *GRHL3* in breast, colon, lung, and skin tissue. Shown are the UMAP (Uniform Manifold Approximation and Projection) plots with the various cell clusters. The corresponding heatmaps are presented to the right of each plot. Data were extracted from the HPA. Scale: light green – low gene expression; red – high gene expression. Note that the three top expressing cell clusters are indicated by a cell-specific and color-coded dot within the heatmaps for both *IRF6* and *GRHL3*. **(B)** Correlations of *IRF6*, *GRHL3*, *CDH1*, and *VIM* within the normal cell lines analyzed in **Figure 1C**. Shown is the Pearson's Correlation Coefficient. Dark green boxes: strong positive correlation; Dark red boxes: strong negative correlation; Light red box: negative correlation.

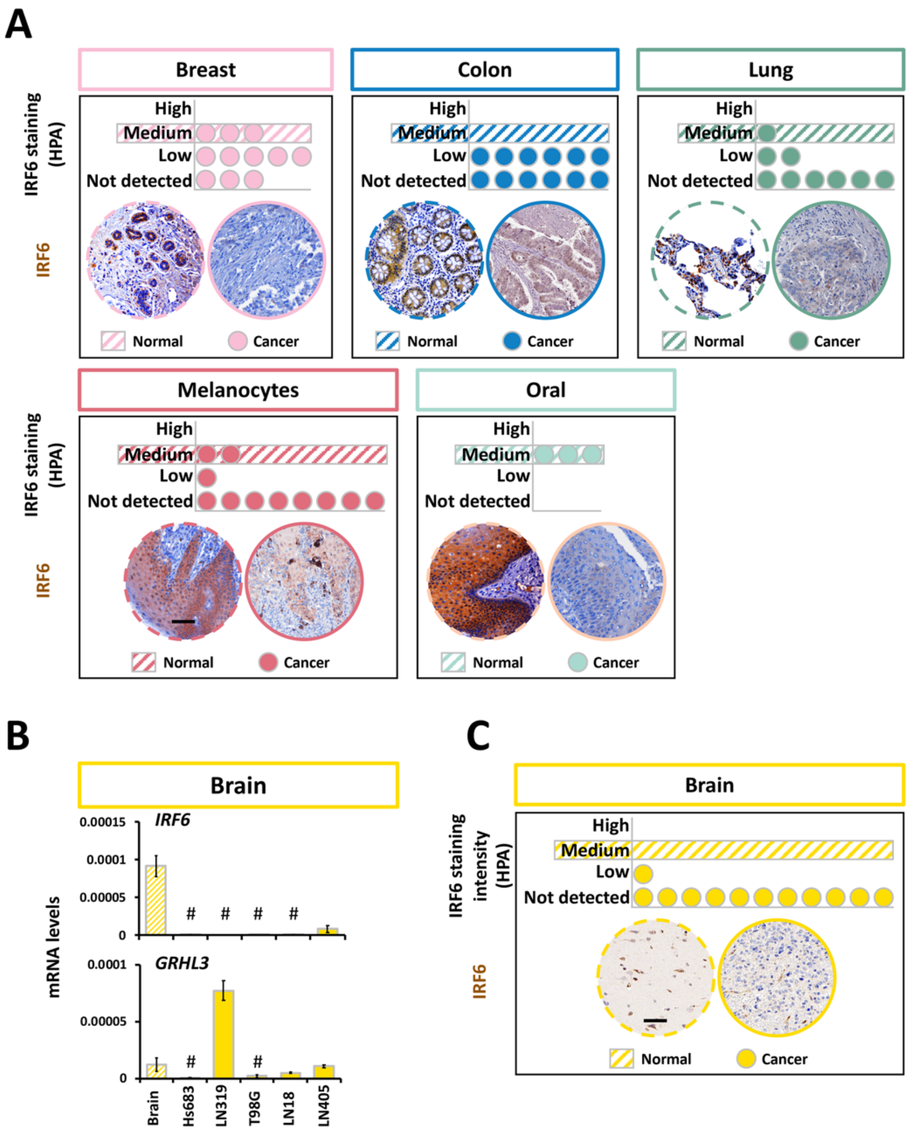

Supplementary Figure 3: Consistent downregulation of IRF6 in carcinomas

(A) The HPA database was used to analyze IRF6 expression in normal and cancerous tissues of the breast, colon, lung, and skin (melanocytes and oral). Expression was quantified as being high, medium, low, and not detected. Each circle represents an individual cancer patient. The striped boxes indicate the IRF6 levels that have been found in the corresponding normal tissues. Note that IRF6 levels in cancer tissue are significantly reduced in breast, colon, lung cancer, and melanoma when compared to their controls. For each tissue type, IHC of normal/cancer tissues is shown for IRF6. Note that IRF6 is readily detectable in the normal tissues, but is mostly absent in the cancer tissues. Scale Bar: 50 µm. **(B)** A panel of brain cancer cell lines was analyzed for the transcript levels of *IRF6* and *GRHL3* compared to brain tissue. # not detectable ($c_T > 32$). **(C)** Only 1/12 brain cancer patients showed low levels of IRF6 while the rest (11/12) was devoid of it. In contrast, IRF6 was found to have a medium staining intensity in normal brain tissue (striped box). Scale Bar: 50 µm. IRF6 staining intensity data collected form the HPA; IHC represents own data.

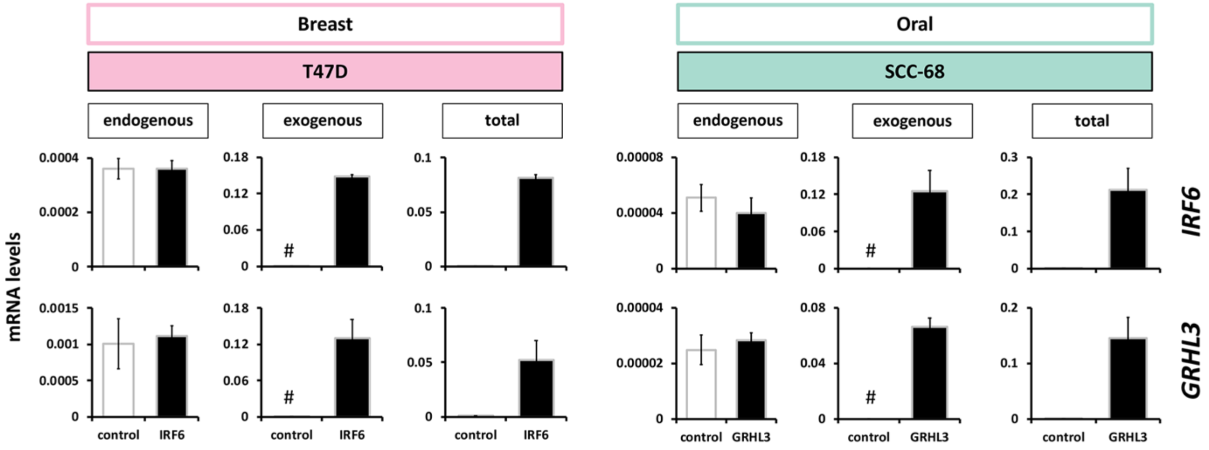

Supplementary Figure 4: Forced expression of IRF6 and GRHL3 in T47D and SCC-68 cell lines

T47D (left) and SCC-68 cell lines were transduced with empty vector control, IRF6, and GRHL3, and *IRF6* and *GRHL3* transcripts analyzed by qPCR. Note that we used specific primer pairs recognizing the endogenous genes only (left panels), the exogenous genes only (middle panels), or both (right panels). # not detectable ($c_T > 32$).

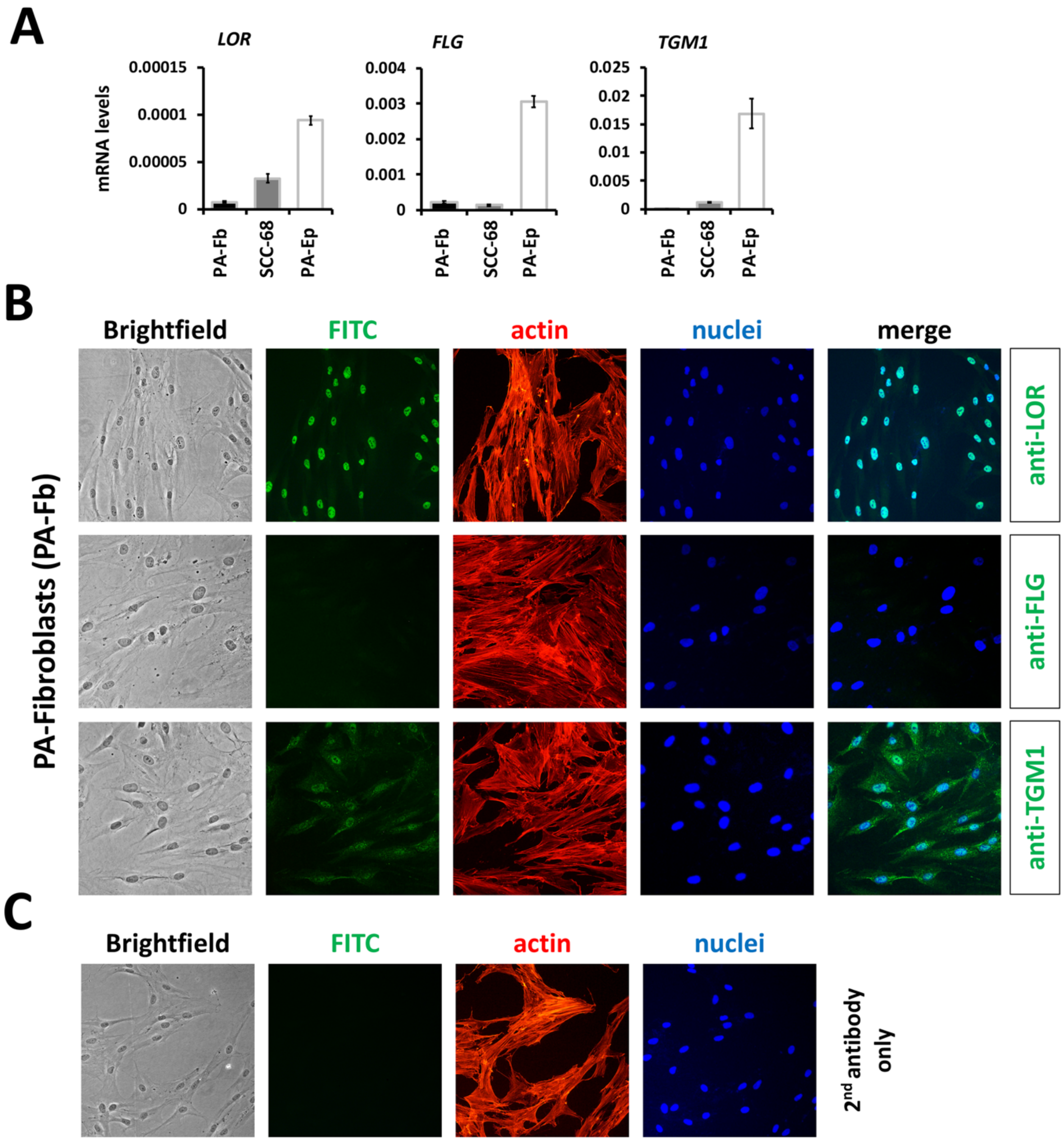

Supplementary Figure 5: Controls for immunofluorescent staining

(A) *LOR*, *FLG*, and *TGM1* mRNA levels were assessed by qPCR in CLP lip fibroblasts (PA-fibroblasts (PA-Fb)), SCC-68, and PA-Ep. Note that the fibroblasts express neglectable levels of all three differentiation markers. **(B)** Staining for LOR, FLG, or TGM1 in PA-Fb revealed nuclear background staining in the FITC channel for LOR and TGM1. **(C)** When only the secondary antibody Alexa Fluor^{TM} 488 goat anti-rabbit IgG (H+L) was used, we did not detect any background fluorescence in the FITC channel.

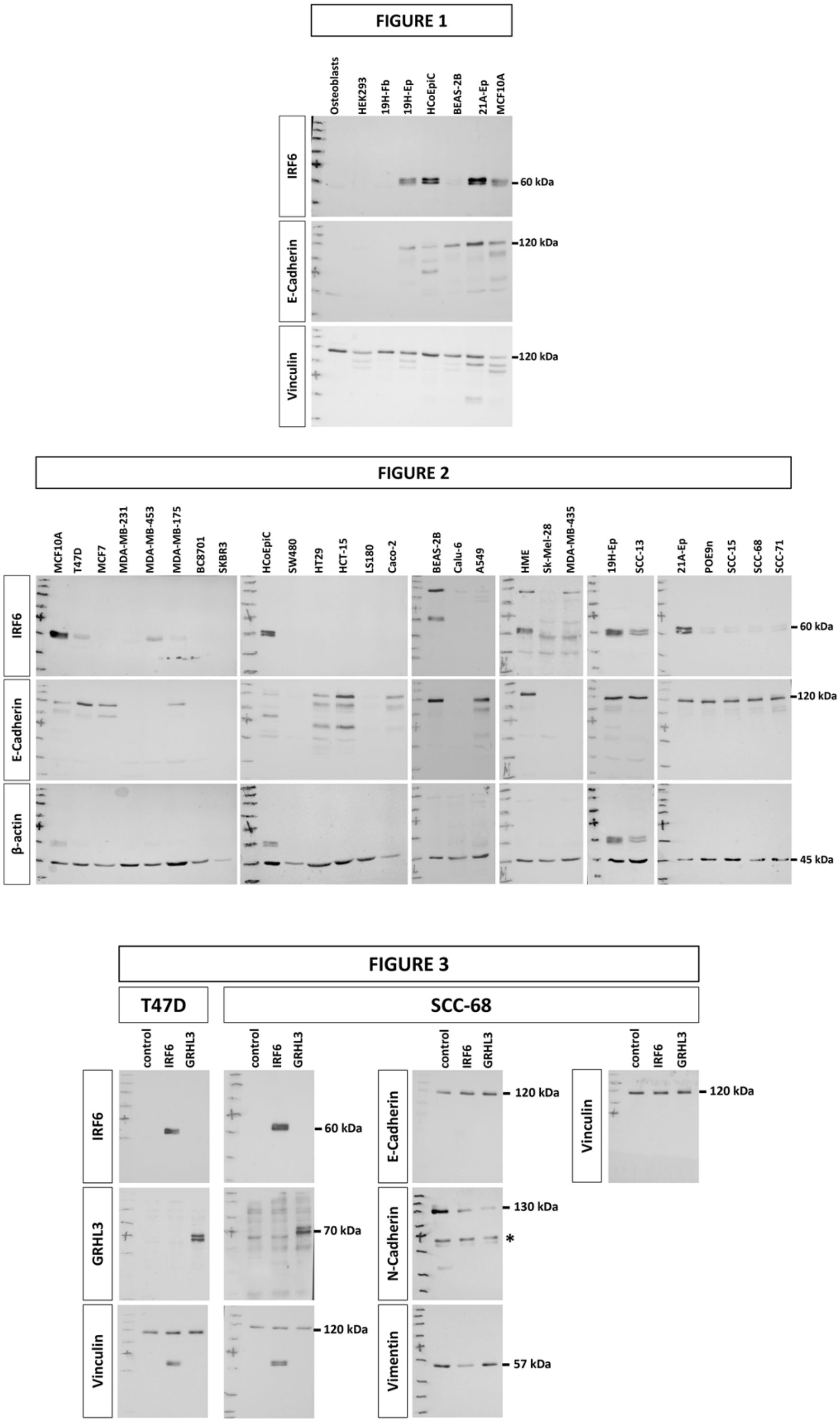

Supplementary Figure 6: Full-length immunoblots

Full-length blots are shown for all the immunoblot analyses performed in this study. HME: human melanocytes. * background band.

Supplementary Table 1: qPCR primers

GENE	SEQUENCE FWD	SEQUENCE REV	AMPLICON (BP)
**Interferon Regulatory Factor 6 *(IRF6)* (endogenous)**	GCTCTTCCATATCATGGCCCTC	CTACAGCCCAGGCCTTAAAAA	200
***IRF6* (exogenous)**	CCTTCAAACCCAGGAGAGCTG	CTGCCAGATCCTCTTCTGAGAT	128
***IRF6* (total)**	CAAGTGTGTGACATCCCTCAG	CCACATCATTATCCFFCTCATCC	91
**Grainyhead-like transcription factor 3 *(GRHL3)* (endogenous)**	ACAGCCTGCACTTCACTGTA	CTCAGATGTGACCTGCCGTC	82
***GRHL3* (exogenous)**	CCCCCACCTGACTGTCTTGA	CTGCCAGATCCTCTTCTGAGAT	199
***GRHL3* (total)**	ACTGTGGAGCACATTGAGGAGG	CTGTGCTCAGACAGTTTACGCC	103
**E-Cadherin 1 *(CDH1)***	AGAACGCATTGCCACATACACT	TCTGATCGGTTACCGTGATCAA	101
**N-Cadherin *(CDH2)***	GGTGGAGGAGAAGAAGACCAG	GGCATCAGGCTCCACAGT	72
**Vimentin *(VIM)***	TGTCCAAATCGATGTGGATGTTTC	TTGTACCATTCTTCTGCCTCCTG	117
**Snail1 *(SNAIL1)***	AAGATGCACATCCGAAGCC	CGCAGGTTGGAGCGGTCAGC	164
TWIST2	CTTATGTTTGGGGGGAGGTT	TAGCCAAGCAATCACGGAGA	295
KI67	TGACTTCCTTCCATTCTGAAGAC	TGGGTCTGTTATTGATGAGCC	109
**Involucrin *(IVL)***	GGCCCTCAGATCGTCTCATA	CACCCTCACCCCATTAAAGA	131
**Filaggrin *(FLG)***	CTGGACACTCAGGTTCCCAT	TTTCGTGTTTGTCTGCTTGC	103
**Loricrin *(LOR)***	AGACCCAGCAGAAGCAGGCG	AGCAGAACTAGATGCAGCCG	200
**Keratin 10 *(K10)***	TGGTTCAATGAAAAGAGCAAGGA	GGGATTGTTTCAAGGCCAGTT	151
**Transglutaminase1 (*TGM1*)**	CCCCCGCAATGAGATCTACA	ATCCTCATGGTCCACGTACACA	73
**Glyceraldehyde 3-phosphate dehydrogenase (*GAPDH*)**	CTCTGACTTCAACAGCGACACCC	TCCTCTTGTGCTCTTGCTGGGGC	199

Sequences (5'-3') of the primer pairs used for the qPCR analyses. FWD: forward; REV: reverse; BP: base pairs.

Supplementary Table 2: Cells

NAME	TISSUE	DISEASE	MORPHOLOGY	MEDIUM	SOURCE
**BMMSC (Bone marrow-derived mesenchyamal stem cells)**	Bone marrow	healthy	fibroblastic	BMMSC	ScienCell
**HUVEC (human umbilical vein endothelial cells)**	Umbilical Cord	healthy	endothelial	1)	Gift from Elizaveta Fasler-Kan, University of Bern, Switzerland
**HEK293 (human embryonic kidney)**	kidney	healthy	epithelial	DMEM	ATCC, CRL-1573
**PA-Ep**	Lip	CLP (wt for *IRF6)*	epithelial	KSFM	Laboratory for Oral Molecular Biology, University of Bern, Switzerland
**E6-Ep**	Lip	CLP (wt for *IRF6)*	epithelial	KSFM	Laboratory for Oral Molecular Biology, University of Bern, Switzerland
**19H-Ep**	Forehead	healthy	epithelial	KSFM	Laboratory for Oral Molecular Biology, University of Bern, Switzerland
**19H-Fb**	Forehead	healthy	fibroblastic	DMEM	Laboratory for Oral Molecular Biology, University of Bern, Switzerland
**21A-Ep**	Gingiva	healthy	epithelial	KSFM	Laboratory for Oral Molecular Biology, University of Bern, Switzerland
**21A-Fb**	Gingiva	healthy	fibroblastic	DMEM	Laboratory for Oral Molecular Biology, University of Bern, Switzerland
**FRSK3-Ep**	Foreskin	healthy	epithelial	KSFM	Laboratory for Oral Molecular Biology, University of Bern, Switzerland
**FRSK3-Fb**	Foreskin	healthy	fibroblastic	DMEM	Laboratory for Oral Molecular Biology, University of Bern, Switzerland
**Wi38**	fetal lung	healthy	fibroblastic	DMEM + NEAA	Gift from Prof. Beat Trüb, University of Bern, Switzerland
**Hepatocytes**	Liver	healthy	epithelial	total RNA	ScienCell (Catalog#5205)
**BEAS-2B**	Lung	healthy	epithelial	KSFM	ATCC, CRL-9609
**HCoEpiC**	Colon	healthy	epithelial	total RNA	ScienCell (Catalog#2955)
**Osteoblasts**	Alveolar Bone	healthy	fibroblastic	DMEM	Laboratory for Oral Molecular Biology, University of Bern, Switzerland
**Melanocytes**	Skin	healthy	stellar, multipolar	1)	ScienCell (Catalog#2205)
**MCF10A**	Mammary Gland	healthy	epithelial	DMEM/F12/Horse serum/EGF/Hydrocortisone, Cholera Toxin, Insulin	ATCC, CRL-10317
**T47D**	Mammary Gland	carcinoma; ductal	epithelial	DMEM	ATCC, HTB-133
**MCF7**	Mammary Gland	adenocarcinoma	epithelial	DMEM	ATCC, HTB-22
**MDA-MB-453**	Mammary Gland	carcinoma; metastatic	epithelial	DMEM	ATCC, HTB-131
**MDA-MB-175**	Mammary Gland	carcinoma; medullary	epithelial	DMEM	ATCC, HTB-25
**MDA-MB-231**	Mammary Gland	adenocarcinoma	epithelial	DMEM	ATCC, CRM-HTB-26
**BC8701**	Mammary Gland	carcinoma; ductal	epithelial	DMEM	Gift from Elizaveta Fasler-Kan, University of Bern, Switzerland
**SKBR3**	Mammary Gland	adenocarcinoma	epithelial	DMEM	ATCC, HTB-30
**SW480**	Colorectal	adenocarcinoma	epithelial	Leibovitz	ATCC, CCL-228
**HT29**	Colorectal	adenocarcinoma	epithelial	McCoys 5A	ATCC, HTB-38
**HCT-15**	Colorectal	adenocarcinoma	epithelial	RPMI-1640	ATCC, CCL-225
**LS180**	Colorectal	adenocarcinoma	epithelial	Eagles MEM	ATCC, CL-187
**Caco-2**	Colorectal	adenocarcinoma	epithelial	DMEM	ATCC, HTB-37
**SW620**	Colorectal	adenocarcinoma	epithelial	Leibovitz	ATCC, CCL-227
**DLD-1**	Colorectal	adenocarcinoma	epithelial	RPMI-1640	ATCC, CCL-221
**Colo 205**	Colorectal	adenocarcinoma	epithelial	RPMI-1640	ATCC, CCL-222
**HCT-116**	Colorectal	carcinoma	epithelial	McCoys 5A	ATCC, CCL-247
**Calu-6**	Lung	carcinoma; anaplastic	epithelial	DMEM	ATCC, HTB-56
**A549**	Lung	carcinoma	epithelial	DMEM	CRM-CCL-185
**Sk-Mel-28**	Skin	malignant melanoma	epithelial	DMEM	ATCC, HTB-72
**MDA-MB-435**	Skin	melanoma	epithelial	DMEM	ATCC, HTB-129
**D10**	Skin	melanoma	epithelial	DMEM	Gift from Elizaveta Fasler-Kan, University of Bern, Switzerland
**SCC-13**	Skin	Squamous Cell Carcinoma	epithelial	KSFM	J. Rheinwald laboratory (Harvard Medical School, Boston, USA)
**POE9n**	oral cavity	Dysplastic Lesion	epithelial	KSFM	J. Rheinwald laboratory (Harvard Medical School, Boston, USA)
**SCC-15**	oral cavity	Oral Squamous Cell Carcinoma	epithelial	KSFM	J. Rheinwald laboratory (Harvard Medical School, Boston, USA)
**SCC-68**	oral cavity	Oral Squamous Cell Carcinoma	epithelial	KSFM	J. Rheinwald laboratory (Harvard Medical School, Boston, USA)
**SCC-71**	oral cavity	Oral Squamous Cell Carcinoma	epithelial	KSFM	J. Rheinwald laboratory (Harvard Medical School, Boston, USA)
**Hs683**	Brain	Oligodendroglioma	epithelial	DMEM	ATCC, HTB-138
**LN319**	Brain	Astrocytoma	epithelial	DMEM	Gift from Jean-Louis Boulay, University of Basel, Switzerland
**T98G**	Brain	Glioblastoma Multiforme	epithelial	DMEM	ATCC, CRL-1690
**LN18**	Brain	Glioblastoma	epithelial	DMEM	Gift from Elizaveta Fasler-Kan, University of Bern, Switzerland
**LN405**	Brain	Glioblastoma	epithelial	DMEM	Gift from Elizaveta Fasler-Kan, University of Bern, Switzerland

Cells used in this study with their names, culture media, and sources. NEAA: non-essential amino acids. 1) only RNA/protein extracts were used. 2) Only RNA was used.

Supplementary Table 3: Literature search strategy

	Keywords	MeSH
**IRF6**	"IFN-regulatory factor 6"<br>"Interferon regulatory factor 6"<br>IRF-6<br>IRF6	"IRF6 protein, human"<br>(Supplementary Concept)
**GRHL3**	"Grainyhead-like 3 protein, human"<br>"Sister-of-Mammalian protein"<br>"Sister-of-Mammalian Grainyhead protein, human"<br>"SOM protein, human"<br>GRHL3<br>GRHL-3<br>"Grainyhead like 3"<br>"Grainyhead-like 3"	"GRHL3 protein, human"<br>(Supplementary Concept)
**Cancer**	Cancer<br>Tumor<br>Tumour*<br>Neoplasm*<br>Carcinoma*<br>Carcinogen*	"Neoplasms"<br>(Mesh)
	**Inclusion**	**Exclusion**
**Criteria**	free access to full text<br>Language: English<br>Discussion of connection to cancer in abstract	Reviews, letters, conference abstracts, book chapters

Table shows the keywords, the MeSH terms as well as inclusion/exclusion criteria for the literature search.

Supplementary Table 4: Literature on the expression/function of IRF6 and GRHL3 in cancer

IRF6

Type	Onc	Sup	Outcome(s)	Ref
**SKIN/ORAL**				
Cutaneous SCC		●	IRF6 downregulation (DNA hypermethylation) correlates with invasiveness and poor differentiation	(1)
		●	IRF6 is a Notch1 target and its downregulation promotes ras-induced tumor formation	(2)
Oral SCC		●	Identification of pathological *IRF6* variants	(3)
	●	●	In response to smoke exposure upregulation of IRF6 that induces NOS2; NOS2 can have dual roles	(4)
		●	Association of an *IRF6* variant with oral SCC	(5)
Vulvar SCC (VSCC)		●	Stepwise downregulation of IRF6 from preneoplastic vulvar lichen sclerosus to VSCC	(6)
Nasorpharyngeal Carcinoma		●	IRF6 downregulation; IRF6 selectively kills cancer stem cells	(7)
Melanoma		●	IRF6 silencing by DNA hypermethylation	(8)
**FEMALE TISSUES**				
Breast Cancer		●	IRF6 downregulation corelating with Maspin	(9)
		●	Re-expression of IRF6 in breast cancer cell induces cell cycle arrest	(10)
	●		IRF6 as a positive regulator of proliferation/differentiation of MCF10A cells downstream of Notch	(11)
	●		ErbB2-driven IRF6 downregulation blocks anoikis and promotes anchorage-independent growth	(12)
		●	IRF6 downregulation in aggressive cancers	(13)
	●		High IRF6 levels after neoadjuvant therapy associated with relapse in ErbB2-positive cancers	(14)
Cervical Cancer		●	Downregulation of IRF6	(15)
Cervical SCC	●		IRF6 negatively correlates with patient survival time	(16)
**GASTROINTESTINAL TRACT**				
Rectal Cancer		●	IRF6 levels inversely associated with rectal cancer	(17)
Colorectal Cancer	●		Positive association of IRF6 and colorectal cancer in relation to smoking/NSAID	(17)
	●		Downregulation of IRF6 as an "anti-cancer effect" of Artemisia annua L. polyphenols (pKAL)	(18)
Gastric Cancer		●	Downregulation of *IRF6* due to DNA methylation of the promoter correlating with poor prognosis	(19)
**RESPIRATORY SYSTEM**				
Lung Cancer	●		Upregulation of IRF6 in asbestos-induced lung cancers	(20)
Large-Cell Lung Cancer; Adenocarcinoma		●	TSCOT, a tumor suppressor is reduced in lung cancer and positively associated with GRHL3 and IRF6	(21)
	●		Upregulation of IRF6 in lung adenocarcinoma and SCC	(22)
**BRAIN**				
Glioma		●	Downregulation of IRF6	(23)
Subependymal Giant Cell Asctrocytoma	●		High expression of IRF6 makes it a potential target for treatment	(24)
**PANCREAS**				
Pancreastic Cancer	●		IRF6 elevated in pancreatic cancer correlting with poor overall survival	(25)
**KIDNEY&URINARY BLADDER**				
Clear Cell Renal Cell Carcinoma		●	Downregulation of IRF6 due to DNA hypermethylation of promoter	(26)
		●	Downregulation of IRF6 that correlates with shorter overall survival of patients	(27)
Urothelial Carcinoma		●	Reexpression of IRF6 inhibits tumor growth; epigenetic regulation of *IRF6*	(28)
**BLOOD**				
Acute Myeloid Leukemia	●		IRF6 overexpression in an AML cell line	(29)

GRHL3

Type	Onc	Sup	Outcome(s)	Ref
**SKIN/ORAL**				
Cutaneous SCC		●	Deletion of GRHL3 evokes loss of PTEN resulting in aggressive tumors	(30)
		●	Decreased GRHL3 contributes to tumor progression	(31)
Oral SCC		●	Loss of GRHL3 promotes tumor by downregulating GSK3B and inducing c-myc	(32)
		●	GRHL3, a negative regulator of PI3K/AKT/mTOR signaling, was downregulated in tumors	(33)
		●	GRHL3-FLG axis upregulation reversed cancer cell proliferation	(34)
Basal-Cell Carcinoma		●	Downregulation of GRHL3; reduces thyroid hormone in the tumor microenvironment	(35)
Skin Cancer (non-melanoma)		●	Reduction of GRHL3 can lead to tumors and inflammatory diseases	(36)
		●	Reduction of GRHL3; GRHL3 a target of oncogenic miR-21	(37)
Trichogerminoma (hair follicle)		●	Identification of GRHL3 rearrangements as oncogenic drivers	(38)
**FEMALE TISSUES**				
Breast Cancer	●		TNFα regulated GRHL3 stimulates endothelial cell migration consistent with an angiogenic fucntion	(39)
	●		ZNF652 represses GRHL3 in normal breast epithelial cells; this response is lost in cancer	(40)
	●	●	Early stage: high expression of GRHL3 (gain of tumor mass); advanced tumors: reduced GRHL3	(41)
		●	Highest GRHL3 levels in stage 1 tumors, lowest in stage 3	(42)
		●	GRHL3 promotes expression of E-Cadherin	(43)
	●		Downregulation of E-Cadherin by GRHL3; induction of migration and invasion by E-Cadherin	(44)
		●	GRHL3 acts downstream of BRD4 to regulate epithelial differentiation; downregulated in tumors	(45)
**GASTROINTESTINAL TRACT**				
Colorectal Cancer	●		Upregulation of GRHL3 in cancer tissue compared to normal tissue	(46)
	●		GRHL3 mRNA and protein levels were higher in cancer tissue than in normal tissue	(47)
	●		GRHL3 promotes tumor growth via MEK signaling	(48)
**RESPIRATORY SYSTEM**				
Small-Cell Lung Cancer; Adenocarcinoma	●		Increased GRHL3 in cancer tissue	(49)
Large-Cell Lung Cancer; Adenocarcinoma		●	The tumor suppressor TSCOT is reduced in lung cancer and positively associated with IRF6	(21)
**PANCREAS**				
Pancreastic Cancer		●	Repression of GRHL3 by SIRT1/CRL4B complex promotes migration and invasion	(51)
**KIDNEY&URINARY BLADDER**				
Renal Carcinoma			No difference between GRHL3 levels in tumors and controls	(52)
Urothelial Carcinoma		●	Overexpression of GRHL3 reduced migration and invasion potential of cancer cells	(53)
**BLOOD**				
Diffuse large B-cell Lymphoma	●		Identification of GRHL3 as a predictor of poor surivival in patients	(54)
**PROXIMAL DIGESTIVE TRACT**				
Esophageal SCC (ESCC)		●	Low GRHL3 levels in P. gingivalis infected ESCC cells stimulates migration/proliferation via PTEN/AKT	(55)

Table indicating the main findings of the studies analyzing expression and/or function of IRF6 (top) and GRHL3 (bottom) in cancer. Onc: oncogene; Sup: Tumor Suppressor.

References Supplementary Table 4

1. Botti E, Spallone G, Moretti F, Marinari B, Pinetti V, Galanti S, et al. Developmental factor IRF6 exhibits tumor suppressor activity in squamous cell carcinomas. Proc Natl Acad Sci U S A. 2011;108(33):13710-5.
2. Restivo G, Nguyen BC, Dziunycz P, Ristorcelli E, Ryan RJ, Ozuysal OY, et al. IRF6 is a mediator of Notch pro-differentiation and tumour suppressive function in keratinocytes. EMBO J. 2011;30(22):4571-85.
3. Stransky N, Egloff AM, Tward AD, Kostic AD, Cibulskis K, Sivachenko A, et al. The mutational landscape of head and neck squamous cell carcinoma. Science. 2011;333(6046):1157-60.
4. Ratovitski EA. DeltaNp63alpha/IRF6 interplay activates NOS2 transcription and induces autophagy upon tobacco exposure. Arch Biochem Biophys. 2011;506(2):208-15.
5. Melath A, Santhakumar GK, Madhavannair SS, Nedumgottil BM, Ramanathan A. A novel heterozygous mutation (F252Y) in exon 7 of the IRF6 gene is associated with oral squamous cell carcinomas. Asian Pac J Cancer Prev. 2013;14(11):6803-6.
6. Rotondo JC, Borghi A, Selvatici R, Magri E, Bianchini E, Montinari E, et al. Hypermethylation-Induced Inactivation of the IRF6 Gene as a Possible Early Event in Progression of Vulvar Squamous Cell Carcinoma Associated With Lichen Sclerosus. JAMA Dermatol. 2016;152(8):928-33.
7. Xu L, Huang TJ, Hu H, Wang MY, Shi SM, Yang Q, et al. The developmental transcription factor IRF6 attenuates ABCG2 gene expression and distinctively reverses stemness phenotype in nasopharyngeal carcinoma. Cancer Lett. 2018;431:230-43.
8. Nobeyama Y, Nakagawa H. Silencing of interferon regulatory factor gene 6 in melanoma. PLoS One. 2017;12(9):e0184444.
9. Bailey CM, Khalkhali-Ellis Z, Kondo S, Margaryan NV, Seftor RE, Wheaton WW, et al. Mammary serine protease inhibitor (Maspin) binds directly to interferon regulatory factor 6: identification of a novel serpin partnership. J Biol Chem. 2005;280(40):34210-7.
10. Bailey CM, Abbott DE, Margaryan NV, Khalkhali-Ellis Z, Hendrix MJ. Interferon regulatory factor 6 promotes cell cycle arrest and is regulated by the proteasome in a cell cycle-dependent manner. Mol Cell Biol. 2008;28(7):2235-43.
11. Zengin T, Ekinci B, Kucukkose C, Yalcin-Ozuysal O. IRF6 Is Involved in the Regulation of Cell Proliferation and Transformation in MCF10A Cells Downstream of Notch Signaling. PLoS One. 2015;10(7):e0132757.
12. Khan IA, Yoo BH, McPhee M, Masson O, Surette A, Dakin-Hache K, et al. ErbB2-driven downregulation of the transcription factor Irf6 in breast epithelial cells is required for their 3D growth. Breast Cancer Res. 2018;20(1):151.
13. Xu HF, Huang TJ, Yang Q, Xu L, Lin F, Lang YH, et al. Candidate tumor suppressor gene IRF6 is involved in human breast cancer pathogenesis via modulating PI3K-regulatory subunit PIK3R2 expression. Cancer Manag Res. 2019;11:5557-72.
14. Surette A, Yoo BH, Younis T, Matheson K, Rameh T, Snowdon J, et al. Tumor levels of the mediators of ErbB2-driven anoikis resistance correlate with breast cancer relapse in patients receiving trastuzumab-based therapies. Breast Cancer Res Treat. 2021;187(3):743-58.
15. Ren Y, Dong J, He P, Liang Y, Wu L, Wang J, et al. miR-587 promotes cervical cancer by repressing interferon regulatory factor 6. J Gene Med. 2020;22(11):e3257.
16. Ding H, Xiong XX, Fan GL, Yi YX, Chen YR, Wang JT, et al. The New Biomarker for Cervical Squamous Cell Carcinoma and Endocervical Adenocarcinoma (CESC) Based on Public Database Mining. Biomed Res Int. 2020;2020:5478574.

17. Slattery ML, Lundgreen A, Bondurant KL, Wolff RK. Interferon-signaling pathway: associations with colon and rectal cancer risk and subsequent survival. Carcinogenesis. 2011;32(11):1660-7.
18. Jung EJ, Paramanantham A, Kim HJ, Shin SC, Kim GS, Jung JM, et al. Identification of Growth Factors, Cytokines and Mediators Regulated by Artemisia annua L. Polyphenols (pKAL) in HCT116 Colorectal Cancer Cells: TGF-beta1 and NGF-beta Attenuate pKAL-Induced Anticancer Effects via NF-kappaB p65 Upregulation. Int J Mol Sci. 2022;23(3).
19. Li D, Cheng P, Wang J, Qiu X, Zhang X, Xu L, et al. IRF6 Is Directly Regulated by ZEB1 and ELF3, and Predicts a Favorable Prognosis in Gastric Cancer. Front Oncol. 2019;9:220.
20. Wright CM, Larsen JE, Hayward NK, Martins MU, Tan ME, Davidson MR, et al. ADAM28: a potential oncogene involved in asbestos-related lung adenocarcinomas. Genes Chromosomes Cancer. 2010;49(8):688-98.
21. Kim KY, Lee G, Yoon M, Cho EH, Park CS, Kim MG. Expression Analyses Revealed Thymic Stromal Co-Transporter/Slc46A2 Is in Stem Cell Populations and Is a Putative Tumor Suppressor. Mol Cells. 2015;38(6):548-61.
22. Liu Y, Shao G, Yang Z, Lin X, Liu X, Qian B, et al. Interferon regulatory factor 6 correlates with the progression of non-small cell lung cancer and can be regulated by miR-320. J Pharm Pharmacol. 2021;73(5):682-91.
23. Lu J, Liu X, Zheng J, Song J, Liu Y, Ruan X, et al. Lin28A promotes IRF6-regulated aerobic glycolysis in glioma cells by stabilizing SNHG14. Cell Death Dis. 2020;11(6):447.
24. Giannikou K, Zhu Z, Kim J, Winden KD, Tyburczy ME, Marron D, et al. Subependymal giant cell astrocytomas are characterized by mTORC1 hyperactivation, a very low somatic mutation rate, and a unique gene expression profile. Mod Pathol. 2021;34(2):264-79.
25. Zhang K, Xu PL, Li YJ, Dong S, Gao HF, Chen LY, et al. Comprehensive analysis of expression profile and prognostic significance of interferon regulatory factors in pancreatic cancer. BMC Genom Data. 2022;23(1):5.
26. Li Z, Yang W, Qiu J, Xu H, Fan B, Li K, et al. Decreased interferon regulatory factor 6 expression due to DNA hypermethylation predicts an unfavorable prognosis in clear cell renal cell carcinoma. J Cancer. 2021;12(22):6640-55.
27. Ma X, Wang X, Dong Q, Pang H, Xu J, Shen J, et al. Inhibition of KIF20A by transcription factor IRF6 affects the progression of renal clear cell carcinoma. Cancer Cell Int. 2021;21(1):246.
28. Jou YC, Lin GL, Lin HY, Huang WH, Chuang YM, Lin RI, et al. Cyproheptadine, an epigenetic modifier, exhibits anti-tumor activity by reversing the epigenetic silencing of IRF6 in urothelial carcinoma. Cancer Cell Int. 2021;21(1):226.
29. Nagel S, Pommerenke C, Meyer C, MacLeod RAF. NKL Homeobox Genes NKX2-3 and NKX2-4 Deregulate Megakaryocytic-Erythroid Cell Differentiation in AML. Int J Mol Sci. 2021;22(21).
30. Darido C, Georgy SR, Wilanowski T, Dworkin S, Auden A, Zhao Q, et al. Targeting of the tumor suppressor GRHL3 by a miR-21-dependent proto-oncogenic network results in PTEN loss and tumorigenesis. Cancer Cell. 2011;20(5):635-48.
31. Bhandari A, Gordon W, Dizon D, Hopkin AS, Gordon E, Yu Z, et al. The Grainyhead transcription factor Grhl3/Get1 suppresses miR-21 expression and tumorigenesis in skin: modulation of the miR-21 target MSH2 by RNA-binding protein DND1. Oncogene. 2013;32(12):1497-507.
32. Georgy SR, Cangkrama M, Srivastava S, Partridge D, Auden A, Dworkin S, et al. Identification of a Novel Proto-oncogenic Network in Head and Neck Squamous Cell Carcinoma. J Natl Cancer Inst. 2015;107(9).
33. Saffarzadeh N, Ghafouri-Fard S, Rezaei Z, Aghazadeh K, Yazdani F, Mohebi M, et al. Expression Analysis of GRHL3 and PHLDA3 in Head and Neck Squamous Cell Carcinoma. Cancer Manag Res. 2020;12:4085-96.

34. Bai Y, Zhao Z, Boath J, van Denderen BJ, Darido C. The functional GRHL3-filaggrin axis maintains a tumor differentiation potential and influences drug sensitivity. Mol Ther. 2021;29(8):2571-82.
35. Di Girolamo D, Ambrosio R, De Stefano MA, Mancino G, Porcelli T, Luongo C, et al. Reciprocal interplay between thyroid hormone and microRNA-21 regulates hedgehog pathway-driven skin tumorigenesis. J Clin Invest. 2016;126(6):2308-20.
36. Hopkin AS, Gordon W, Klein RH, Espitia F, Daily K, Zeller M, et al. GRHL3/GET1 and trithorax group members collaborate to activate the epidermal progenitor differentiation program. PLoS Genet. 2012;8(7):e1002829.
37. Kikulska A, Rausch T, Krzywinska E, Pawlak M, Wilczynski B, Benes V, et al. Coordinated expression and genetic polymorphisms in Grainyhead-like genes in human non-melanoma skin cancers. BMC Cancer. 2018;18(1):23.
38. Kervarrec T, Pissaloux D, Poilane J, Tirode F, Tallet A, Collin C, et al. Recurrent FOXK1::GRHL and GPS2::GRHL fusions in trichogerminoma. J Pathol. 2022;257(1):96-108.
39. Guardiola-Serrano F, Haendeler J, Lukosz M, Sturm K, Melchner H, Altschmied J. Gene trapping identifies a putative tumor suppressor and a new inducer of cell migration. Biochem Biophys Res Commun. 2008;376(4):748-52.
40. Kumar R, Selth LA, Schulz RB, Tay BS, Neilsen PM, Callen DF. Genome-wide mapping of ZNF652 promoter binding sites in breast cancer cells. J Cell Biochem. 2011;112(10):2742-7.
41. Panis C, Pizzatti L, Herrera AC, Cecchini R, Abdelhay E. Putative circulating markers of the early and advanced stages of breast cancer identified by high-resolution label-free proteomics. Cancer Lett. 2013;330(1):57-66.
42. Xu H, Liu C, Zhao Z, Gao N, Chen G, Wang Y, et al. Clinical implications of GRHL3 protein expression in breast cancer. Tumour Biol. 2014;35(3):1827-31.
43. Alotaibi H, Basilicata MF, Shehwana H, Kosowan T, Schreck I, Braeutigam C, et al. Enhancer cooperativity as a novel mechanism underlying the transcriptional regulation of E-cadherin during mesenchymal to epithelial transition. Biochim Biophys Acta. 2015;1849(6):731-42.
44. Zhao P, Guo S, Tu Z, Di L, Zha X, Zhou H, et al. Grhl3 induces human epithelial tumor cell migration and invasion via downregulation of E-cadherin. Acta Biochim Biophys Sin (Shanghai). 2016;48(3):266-74.
45. Nagarajan S, Bedi U, Budida A, Hamdan FH, Mishra VK, Najafova Z, et al. BRD4 promotes p63 and GRHL3 expression downstream of FOXO in mammary epithelial cells. Nucleic Acids Res. 2017;45(6):3130-45.
46. Wang XK, Zhou FF, Tao HR, Wang X, Zhang C, Su F, et al. Knockdown of GRHL3 inhibits activities and induces cell cycle arrest and apoptosis of human colorectal cancer cells. J Huazhong Univ Sci Technolog Med Sci. 2017;37(6):880-5.
47. Yuan M, Wang J, Fang F. Grainyhead-Like Genes Family May Act as Novel Biomarkers in Colon Cancer. Onco Targets Ther. 2020;13:3237-45.
48. Tan L, Qu W, Wu D, Liu M, Wang Q, Ai Q, et al. GRHL3 Promotes Tumor Growth and Metastasis via the MEK Pathway in Colorectal Cancer. Anal Cell Pathol (Amst). 2021;2021:6004821.
49. Taniwaki M, Daigo Y, Ishikawa N, Takano A, Tsunoda T, Yasui W, et al. Gene expression profiles of small-cell lung cancers: molecular signatures of lung cancer. Int J Oncol. 2006;29(3):567-75.
50. Leng S, Huang W, Chen Y, Yang Y, Feng D, Liu W, et al. SIRT1 coordinates with the CRL4B complex to regulate pancreatic cancer stem cells to promote tumorigenesis. Cell Death Differ. 2021;28(12):3329-43.

51. Pawlak M, Kikulska A, Wrzesinski T, Rausch T, Kwias Z, Wilczynski B, et al. Potential protective role of Grainyhead-like genes in the development of clear cell renal cell carcinoma. Mol Carcinog. 2017;56(11):2414-23.
52. Wezel F, Lustig J, Azoitei A, Liu J, Meessen S, Najjar G, et al. Grainyhead-Like 3 Influences Migration and Invasion of Urothelial Carcinoma Cells. Int J Mol Sci. 2021;22(6).
53. Liu W, Ha M, Wang X, Yin N. Clinical significance of GRHL3 expression in diffuse large B cell lymphoma. Tumour Biol. 2016;37(7):9657-61.
54. Liang G, Wang H, Shi H, Zhu M, An J, Qi Y, et al. Porphyromonas gingivalis Promotes the Proliferation and Migration of Esophageal Squamous Cell Carcinoma through the miR-194/GRHL3/PTEN/Akt Axis. ACS Infect Dis. 2020;6(5):871-81.

Supplementary Movie 1: Migration of SCC-68/control cells

Confluent cells were scratched with a pipet tip and cell free gap was monitored using the IncuCyte S3 live imaging device. Pictures were taken at 0, 2, 4, 6, 8, and 10 h after scratching the cell monolayer.

Supplementary Movie 2: Migration of SCC-68/IRF6 cells

Confluent cells were scratched with a pipet tip and cell free gap was monitored using the IncuCyte S3 live imaging device. Pictures were taken at 0, 2, 4, 6, 8, and 10 h after scratching the cell monolayer. Note that the SCC68/IRF6 cells display a significant delay in the closure of the gap when compared to control.

Supplementary Movie 3: Migration of SCC-68/GRHL3 cells

Confluent cells were scratched with a pipet tip and cell free gap was monitored using the IncuCyte S3 live imaging device. Pictures were taken at 0, 2, 4, 6, 8, and 10 h after scratching the cell monolayer. Note that the SCC-68/GRHL3 cells display a significant delay in the closure of the gap when compared to control.
